# Comprehensive Assessment of Biochemical Traits in Diverse Faba Bean (*Vicia faba* L.) Germplasm for Selection of Superior Accessions

**DOI:** 10.1002/fsn3.70931

**Published:** 2025-09-08

**Authors:** Kebede Taye Desta, Myoung‐Jae Shin, Sukyeung Lee, Hyemyeong Yoon, Jungyoon Yi, Heon‐Woong Kim, Yu‐Mi Choi

**Affiliations:** ^1^ National Agrobiodiversity Center National Institute of Agricultural Sciences, Rural Development Administration Jeonju Republic of Korea; ^2^ International Technology Cooperation Center, Technology Cooperation Bureau Rural Development Administration Jeonju Republic of Korea; ^3^ Department of Agrofood Resources, Food and Nutrition Division National Institute of Crop and Food Science, Rural Development Administration Wanju‐gun Republic of Korea

**Keywords:** antinutrients, antioxidant activity, diversity, foods, legume biochemicals

## Abstract

This study evaluated the diversity of 68 faba bean accessions grown in the Republic of Korea, focusing on variations in antinutrient factors, nutritional composition, fatty acids, lipid quality, antioxidant activity, and key agronomical traits. All parameters showed significant variations, indicating broad genetic diversity. Vicine and convicine were detected in all accessions using UHPLC–MS/MS, with their concentrations varying more than threefold. Total phenol (1.65–4.38 mg GAE/g), saponin (4.12–9.50 mg DE/g), and tannin (1.70–9.47 mg CE/g) contents showed coefficients of variation (CV) above 18.00%. Similarly, crude fiber (3.42%–10.85%), dietary fiber (9.18%–25.47%), total protein (21.68%–34.40%), and total fat (0.90%–1.99%) contents exhibited wide variations (CV > 9.50%). Palmitic acid (15.13%–18.73%) and linoleic acid (38.51%–54.93%) were the dominant saturated and unsaturated fatty acids, respectively. Antioxidant activities, including DPPH^•^ scavenging activity (0.40–2.33 mg AAE/g), ABTS^•+^ scavenging activity (1.87–7.26 mg TE/g), and ferric reducing antioxidant power (0.67–4.94 mg AAE/g) varied significantly, all with CVs over 25.00%. Green genotypes exhibited significantly higher tannin, fibers, and palmitic and total saturated fatty acids, while yellow genotypes showed higher antioxidant activities, total unsaturated fatty acids, and lipid quality indices. Cluster and principal component analyses differentiated accessions based on their overall biochemical profile. Accession VF050 was identified for its low antinutrient content, VF012 and VF004 for distinct antioxidant potential, VF062, VF027, and VF012 for lipid quality, VF032, VF009, and VF018 for high fiber, VF041 and VF057 for high protein, and VF019 and VF027 for agronomic performance. These accessions could serve as valuable materials for food innovation, crop improvement, and cultivation.

## Graphical abstract

A total of 68 faba bean accessions cultivated in Korea were assessed for their biochemical and agronomic diversity, revealing significant variations in antinutrient factors, nutrient composition, and antioxidant activity. Green genotypes tended to have higher tannin content, fiber levels, and saturated fatty acids, whereas yellow genotypes exhibited enhanced antioxidant activity and superior lipid quality. Based on these findings, specific accessions were identified as promising candidates for food innovation and genetic improvements.

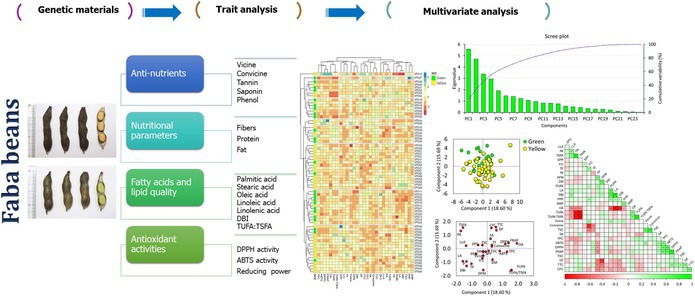

## 1 Introduction

Faba bean ( *Vicia faba* L.) is a widely known legume within the genus *Vicia*. The crop is becoming popular for its value in human nutrition, animal feed, and sustainable agriculture (Millar et al. 2019; Nurgi et al. 2023; Vilariño et al. 2009). The beans are rich in essential nutrients, including fiber, fatty acids, carbohydrates, and minerals. They are also good sources of plant‐based protein and play a crucial role in global food security strategies and vegan diets (Fasakin et al. 2024; Hamed et al. 2024; Meng et al. 2021; Moguel‐Concha et al. 2023; Shi et al. 2024). In addition, faba beans are rich in numerous classes of secondary metabolites, including phenolic acids, lignans, flavonoids, and anthocyanins. These compounds are known for their antioxidant, anti‐inflammatory, and anticancer properties, and contribute to disease prevention (Feng et al. 2024; Shi et al. 2024; Yehmed et al. 2024).

Despite their nutritional benefits and health‐promoting compounds, faba beans contain antinutrients such as vicine, convicine, tannins, saponins, and phenols. These metabolites can interfere with nutrient digestion and absorption (Gutierrez and Torres 2019; Khazaei et al. 2019; Singh et al. 2023). Consuming faba beans with a high concentration of these biochemical components also results in adverse reactions, including favism, in people with certain sensitivities (Khazaei et al. 2019). Moreover, the presence of high levels of tannins and saponins results in an undesirable flavor (Shi et al. 2024; Singh et al. 2023). All these factors limit the consumption of faba beans and their widespread use in the food industry. Therefore, understanding the balance between beneficial metabolites and antinutrients is essential to enhancing the health benefits of faba beans while minimizing their negative effects (Khamassi et al. 2013; Lippolis et al. 2025; Salvador‐Reyes et al. 2024).

To fully explore the potential of faba beans, it is crucial to understand their overall biochemical diversity as highlighted before. This necessitates studies focusing on the biochemical analysis of faba bean genetic materials, as well as the investigation of how genetic factors, morphological traits, environmental conditions, and post‐harvest practices affect their biochemical makeup (Göl et al. 2017; Jha et al. 2024; Walter et al. 2023; Yilmaz and Yilmaz 2025). Beyond understanding the basic compositions and their diversity, such studies support the selection of materials with desirable traits and lead to the development of varieties with high nutritional quality, lower antinutrient levels, and improved disease resistance through breeding (Akgun and Canci 2023; Göl et al. 2017; Gutierrez and Torres 2019; Yilmaz and Yilmaz 2025). A deep understanding of the biochemical diversity of faba bean genetic materials is also key to developing functional foods that offer maximized health benefits (Thomsen et al. 2025).

In the Republic of Korea, the application of faba beans in the food industry and local agriculture is in the early stages. Moreover, progress in their development is slower than that of other legumes such as soybeans and mung beans, and no local cultivars have been developed thus far. Despite this, public interest in their dietary use is steadily growing. Accordingly, exploring the genetic diversity of available resources in terms of both agronomic performance and biochemical composition is essential to address the growing demand and advance the development of improved varieties (Hamed et al. 2024; Salvador‐Reyes et al. 2024; Walter et al. 2023). This study aimed to conduct a comprehensive evaluation of 68 faba bean genetic resources cultivated in the Republic of Korea, focusing on their antinutrient factors, nutritional composition, fatty acid profiles, lipid quality, and antioxidant activities. Additionally, the influence of seed coat color variation on these biochemical traits was statistically analyzed. The study could provide valuable insights into the biochemical diversity within the faba bean germplasm and help identify superior genotypes with desirable nutritional and functional properties. These findings of this study could have potential applications in food processing, crop improvement programs, and dissemination to farmers for enhanced cultivation practices.

## 2 Materials and Methods

### 2.1 Faba Bean Seeds, Chemicals, and Materials

A total of 68 faba bean germplasm was obtained from the 2021 cultivation season at the National Agrobiodiversity Center, Jeonju, Republic of Korea. These genetic materials were grown under uniform field conditions. Seed samples from each accession were freeze‐dried, ground into a fine powder, sieved, and stored at −20°C until further analysis. Based on seed coat color, the accessions were grouped as green (*n* = 26) and yellow (*n* = 42) genotypes. The list of all accessions, along with their basic information, is presented in Table S1. All reagents and chemicals used in this study were of analytical grade. Convicine standard was obtained from Toronto Research Chemicals (Toronto, ON, Canada). Ethanol and sulfuric acid were purchased from Fisher Scientific (Pittsburgh, PA, USA). All the remaining reference standards, chemicals, and reagents were purchased from Sigma‐Aldrich (St. Louis, MO, USA). For sample preparation, centrifuge tubes from SPL Life Sciences (Pocheon‐si, Gyeonggi‐do, Korea) and round‐bottom glass tubes with screw caps from SciLab (Seoul, Korea) were used. A Labogene 1236R centrifuge (Labogene, Seoul, Korea) was used for centrifugation at 3134× *g*. Colorimetric assays were conducted using 96‐well plates obtained from Thermo Fisher Scientific (Waltham, MA, USA) and absorbance was measured using an Eon Microplate Spectrophotometer (Bio‐Tek, Winooski, VT, USA). Ultra‐high‐performance liquid chromatography‐diode array detector (UPLC‐DAD) system coupled to quadrupole time‐of‐flight (QToF) mass spectrometry (SCIEX X500R, SCIEX Co., Framingham, MA, USA) was used for vicine and convicine analyses. A CORTECS UPLC T3 column (2.1 × 150 mm, 1.6 μm, Waters Co., Milford, MA, USA) and a CORTECS UPLC Vanguard T3 column were used for separation. Fiber and total fat content were measured using an Analytical Fibertec system and a Soxtec extractor, respectively (FOSS, Hillerød, Denmark). Likewise, a QP2010 gas chromatography‐flame ionization detector (GC‐FID) instrument (Shimadzu, Kyoto, Japan) equipped with an HP‐INNOWAX column (30 m × 0.250 mm, 0.25 μm) was used for the analysis of fatty acids.

### 2.2 UPLC‐MS/MS Analysis of Vicine and Convicine

Extraction of vicine and convicine was conducted using 7% perchloric acid as an extraction solvent (Pulkkinen et al. 2015). Briefly, 0.50 g of powdered sample was mixed with 7.5 mL of the solvent, vortexed for 1 min, and centrifuged for 10 min at 4°C. The supernatant was collected, and the process was repeated with the remaining residue. The supernatants were combined, filtered, and transferred to an injection vial for analysis. The structures of vicine and convicine were identified using a UPLC‐DAD‐MS/MS system in a positive electrospray ionization (+ESI) mode within an m/z range of 100–1200. The ion source gas, curtain gas, and ion source temperature were set to 50 psi, 30 psi, and 450°C, respectively. Likewise, declustering potential, collision energy, and ion spray voltage were 80, 15 ± 10, and 5500 V, respectively. A binary solvent system of water (A) and acetonitrile with 0.1% formic acid (B) was used as a mobile phase at a flow rate of 0.8 mL/min. Initially, an isocratic run with 100% solvent A was maintained for 15 min, after which solvent B was linearly increased to 70% for 7 min. The latter was then held constant for 3 min, and the post‐run time was set at 10 min. The sample injection volume was 5 μL. Chromatographic detection of both compounds was performed at 273 nm. Calibration curves plotted from vicine and convicine external standards were used for quantification, and results were determined in milligrams per gram of dry seed weight (mg/g) from three replicate measurements.

### 2.3 Determination of Total Metabolite Contents

The total tannin, saponin, and phenol contents were determined using a previously outlined method (Boudjou et al. 2013). Initially, 0.5 g of powdered sample was mixed with 5 mL of 80% ethanol in a 15 mL extraction tube, sonicated in the dark for 45 min, and centrifuged. The supernatant was collected, and the residue was extracted again. The combined supernatants were used for analysis in a 96‐well microplate reader. The total tannin content was measured using the vanillin‐HCl method, and catechin was used as a standard (Price et al. 1978). Likewise, the total saponin content was determined by the vanillin‐sulfuric acid assay using diosgenin as a standard (Boudjou et al. 2013), while the total phenol content was measured using the Folin–Ciocalteu method, using gallic acid as a standard (Choi et al. 2024). The results of each were expressed as milligrams equivalent of the standard compounds per gram of dried seed weight.

### 2.4 Determination of Total Protein, Fat, and Fiber Contents

Crude fiber and dietary fiber contents were measured using AOAC methods (AOAC 1990). The crude fiber content was assessed using a fiber analyzer, while dietary fiber content was measured with an enzymatic‐gravimetric assay. Crude protein content was determined using the Kjeldahl method and calculated as *N* × 6.25. Total fat content was measured using Soxhlet extraction with n‐hexane. The contents of all parameters were expressed as percentages on the basis of dried seed weight.

### 2.5 GC‐FID Analysis of Fatty Acids

Fatty acid analysis was conducted by preparing fatty acid methyl esters using a direct methylation method (Choi et al. 2024). During GC‐FID analysis, a 1 μL injection volume was used with a 50:1 split ratio. The carrier gas was helium at a flow rate of 1.5 mL/min. The column temperature was initially set at 100°C, then increased to 170°C at a rate of 60°C/min, with a 1‐min hold time. It was further raised to 240°C at a rate of 6.5°C/min and held for another minute, making the total analysis time 16.41 min. The temperatures of the detector and injection port were both set to 250°C. Fatty acids were identified by comparing their retention times with external standards. The contents of each fatty acid were determined as a percentage based on chromatogram peak area response. Total saturated fatty acid (TSFA) was determined as the sum of palmitic acid [16:0] and stearic acid [18:0], total unsaturated fatty acid (TUFA) as the sum of oleic acid [18:1], linoleic acid [18:2], and linolenic acid [18:3], and polyunsaturated fatty acid (PUFA) as the sum of linoleic acid and linoleic acid. Oleic acid was the only monounsaturated fatty acid (MUFA). The overall oil stability was estimated using the TUFA‐to‐TSFA ratio and double bond index (DBI), the latter being determined using the following Equation (1) (Tilami and Kouřimská 2022).

(1) $$\begin{array}{cc} \mathrm{DBI}=\; & 0\times \left(\left[14:0\right]+\left[15:0\right]+\left[16:0\right]+\left[18:0\right]+\left[20:0\right]\right)\\ & +1\times \left(\left[16:1\right]+\left[18:1\right]+\left[20:1\right]\right)+2\times \left(\left[18:2\right]+\left[20:2\right]\right)\\ & +3\times \left(\left[18:3\right]\right)+4\times \left(\left[20:4\right]\right) \end{array}$$

### 2.6 Determination of Antioxidant Activities

The extraction technique used for total tannin, saponin, and phenol analysis was also applied to assess all antioxidant activities. During each assay, measurement was performed in triplicate using a 96‐well microplate (Choi et al. 2024). DPPH^•^ scavenging activity and ferric‐reducing antioxidant power (FRAP) were determined as milligrams of ascorbic acid equivalents per gram of dried seed weight (mg AAE/g) using Equation (2). ABTS^•+^ scavenging activity was determined in milligrams of Trolox equivalents per gram of dried seed weight (mg TE/g).

(2) $$\text{Antioxidant capacity}\;\left(\mathrm{mg}\;\mathrm{STD}\;g^{-1}\mathrm{DW}\right)=\frac{\left(C\times V\right)}{m}\times \mathrm{Df}$$

In the above equation, C, V, Df, and m represent the concentration (mg/mL) of the sample corresponding to the calibration curve of the standard (STD), total sample volume (mL), dilution factor, and sample weight (g), respectively.

### 2.7 Statistical Analysis

In this study, all measurements and analyses were performed in triplicate and results are presented as mean ± standard deviation (SD). Analysis of variance (ANOVA) followed by Duncan's multiple range test at *p* < 0.05 was used to determine significant differences between measurements using XLSTAT software version 2019.2.2 (Lumivero, CO, USA). Principal component analysis (PCA) was computed using JMP software version 17 (SAS Inc., Cary, NC, USA). Additionally, boxplots, two‐way hierarchical cluster analysis (HCA), and Pearson's correlation analyses were performed using R software version 4.0.2 (R‐Project).

## 3 Results

### 3.1 Agro‐Morphological Characteristics of Faba Bean Accessions

**FIGURE 1:**
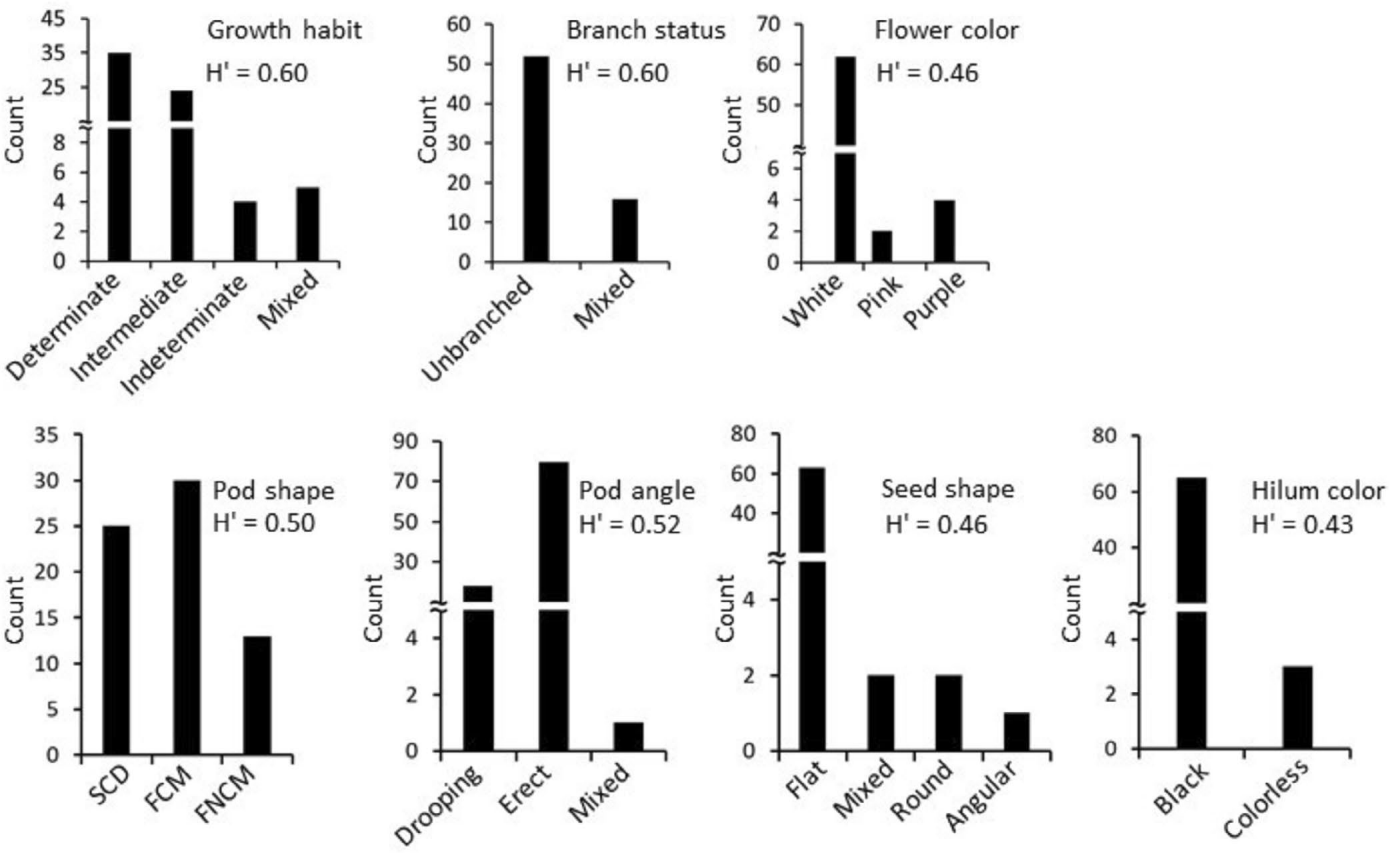
Frequency distribution and diversity index of categorical agronomic traits across 68 faba bean accessions.

**FIGURE 2:**
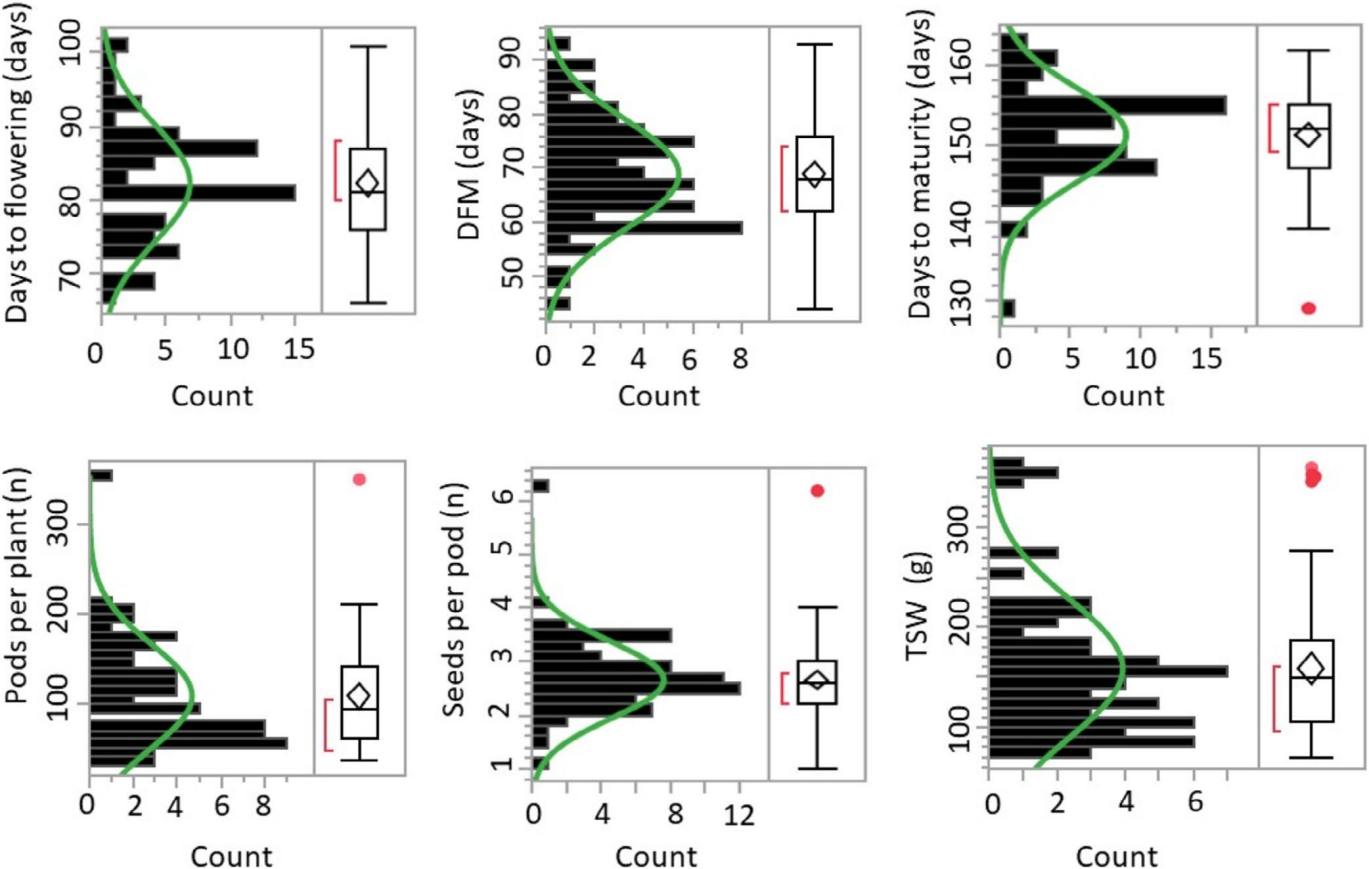
Histograms and boxplots showing the distributions of quantitative agronomical traits across 68 faba bean accessions. In the boxplots, upper and lower edges represent 75th percentile and 25th percentile, respectively, whiskers extend 1.5× the interquartile ranges, central horizontal lines represent median values, and values outside whiskers indicate outliers.

The distribution of categorical traits is shown in Figure 1, while their frequency values are presented in Table S2. Growth habit and pod shape recorded the highest standardized Shannon‐Weaver index (H′) value (0.60). Accordingly, most of the faba bean accessions exhibited a determinate growth habit (51.47%), followed by an intermediate type (35.29%). The most common pod shape was flat with a concave middle (44.12%), followed by the cylindrical type (36.72%). Branch status and pod angle also showed relatively high diversity, each with an H′ value of ≥ 0.50. In contrast, flower color, hilum color, and seed shape had lower H′ values (≤ 0.46) and were dominated by a single characteristic (Table S2). Quantitative agronomical traits also showed wide variations (Figure 2, Table S3). Days to flowering (DF), days from flowering to maturity (DFM), and days to maturity (DM) were in the ranges of 66 to 101, 44 to 93, and 129 to 162 days, with means of 82.28, 68.87, and 151.15 days, respectively. Accession VF041 was the earliest to flower, while VF015 was the earliest to mature. In contrast, VF008 and VF012 both showed the longest DF, while VF062 recorded the longest DM. Regarding yield‐related traits, seeds per pod (SPP) ranged from 1.00 to 6.20, pods per plant (PPP) from 35.00 to 351.00, and seed weight per plant (SWP) from 71.00 to 360.00 g, with coefficients of variation (CV) of 53.25%, 26.63%, and 43.05%, respectively. The highest SPP was observed in accession VF049, whereas VF027 recorded the highest PPP and SWP. In contrast, accessions VF006, VF007, and VF034 had the lowest SPP, PPP, and SWP, respectively.

### 3.2 Antinutrient Parameters

The antinutrient contents of the faba bean accessions are presented in Table 1, while a statistical summary is provided in Table S4. All parameters showed significant variations between the accessions (*p* < 0.05), with a CV ranging from 16.29% for convicine to 37.11% for total tannin content. Accession VF009 recorded the highest vicine content (12.68 mg/g), which was significantly different from all other accessions, except for accession VF005. Accession VF028 exhibited significantly higher convicine (9.74 mg/g) and total vicine‐convicine (TVC) content (19.34 mg/g) compared to all other accessions, except four and five specific accessions, respectively (Table 1). Accessions VF040, VF066, and VF050 had the lowest vicine (3.41 mg/g), convicine (1.75 mg/g), and TVC (9.19 mg/g), respectively. The lowest vicine‐to‐convicine ratio was found in VF040 (0.42) and the highest in VF066 (5.67). The highest total tannin observed in accession VF018 (9.47 mg CE/g) was also significantly different from all other accessions. Conversely, accession VF052 had the lowest total tannin content (1.63 mg CE/g). The highest total phenolic content was recorded in accession VF036 (4.38 mg GAE/g), significantly higher than all other accessions, except for VF035. Accession VF057 exhibited the highest total saponin content (9.50 mg DE/g), which was significantly different from the other accessions, except four specific accessions (Table 1). In contrast, accessions VF032 and VF003 recorded the lowest total phenol (1.65 mg GAE/g) and total saponin (4.12 mg DE/g) contents, respectively.

**TABLE 1:** Variations of vicine, convicine, TVC, total tannin, total phenol, and total saponin contents among 68 faba bean accessions.

IT Number	Accession	Convicine (mg/g)	Vicine (mg/g)	TVC (mg/g)	TPC (mg GAE/g)	TTC (mg CE/g)	TSC (mg DE/g)
138252	VF001	9.39 ± 0.60 a–b	8.19 ± 0.45 u–x	17.58 ± 1.03 d–i	2.99 ± 0.10 g–m	2.88 ± 0.23 s–z	4.63 ± 0.44 u–x
161022	VF002	5.59 ± 0.14 j–m	6.60 ± 0.04 a–f	12.19 ± 0.13 t–x	2.65 ± 0.13 j–u	3.50 ± 0.44 p–u	4.53 ± 0.42 u–x
163348	VF003	5.51 ± 0.12 j–m	6.92 ± 0.12 ad–af	12.43 ± 0.15 r–x	2.71 ± 0.16 j–s	3.66 ± 0.07 n–s	4.12 ± 0.20 x
188090	VF004	6.50 ± 0.13 hi	10.60 ± 0.07 d–g	17.10 ± 0.10 g–j	3.84 ± 0.19 b,c	3.57 ± 0.51 o–u	6.26 ± 0.57 j–t
200130	VF005	5.75 ± 0.18 j–l	12.54 ± 0.12 a	18.28 ± 0.26 b–f	2.33 ± 0.18 r–w	4.11 ± 0.33 m–q	5.28 ± 0.28 r–x
203426	VF006	8.89 ± 0.24 b,c	7.76 ± 0.07 x–aa	16.64 ± 0.18 i,j	2.57 ± 0.01 L–v	4.15 ± 0.23 L–p	7.17 ± 0.98 e–m
203427	VF007	6.64 ± 0.18 g–h	6.56 ± 0.06 af	13.20 ± 0.20 o–u	2.49 ± 0.04 o–v	2.48 ± 0.10 w–ab	6.11 ± 0.40 k–t
208231	VF008	3.88 ± 0.17 q–t	9.81 ± 0.20 j–o	13.69 ± 0.37 n–q	2.86 ± 0.15 h–o	6.07 ± 0.49 c–d	5.30 ± 0.09 r–x
208235	VF009	4.14 ± 0.21 p–r	12.68 ± 0.21 a	16.82 ± 0.40 i,j	2.99 ± 0.01 g–m	4.93 ± 0.16 g–l	7.04 ± 0.67 e–n
208722	VF010	3.12 ± 0.15 u–x	7.66 ± 0.26 y–ab	10.79 ± 0.37 y–aa	2.60 ± 0.14 k–u	5.12 ± 0.16 e–j	6.94 ± 0.39 e–o
208723	VF011	4.16 ± 0.32 p–r	8.85 ± 0.44 q–r	13.01 ± 0.76 p–u	2.57 ± 0.06 L–v	6.65 ± 0.73 b,c	6.53 ± 0.35 g–s
208724	VF012	3.60 ± 0.08 r–w	7.78 ± 0.45 w–aa	11.38 ± 0.52 x–z	3.74 ± 0.08 b–d	7.22 ± 0.08 b	7.07 ± 0.72 e–n
208728	VF013	3.62 ± 0.12 r–v	6.96 ± 0.04 ac–af	10.58 ± 0.14 z–aa	2.67 ± 0.03 j–t	4.55 ± 0.25 h–m	7.67 ± 0.36 c–j
212781	VF014	3.63 ± 0.18 r–v	8.50 ± 0.12 q–u	12.13 ± 0.31 u–x	2.66 ± 0.10 j–t	5.11 ± 0.41 e–j	5.82 ± 0.85 L–u
228951	VF015	8.79 ± 0.35 b–d	8.77 ± 0.06 q–t	17.56 ± 0.41 d–i	2.24 ± 0.06 t–x	5.21 ± 0.34 e–i	7.03 ± 0.42 e–n
K013509	VF016	6.48 ± 0.13 h,i	10.26 ± 0.12 f–j	16.74 ± 0.21 i,j	2.99 ± 0.02 g–m	5.45 ± 0.04 d–g	6.87 ± 0.60 e–p
K014965	VF017	4.78 ± 1.70 n–p	8.30 ± 0.40 s–w	13.08 ± 2.10 o–u	2.61 ± 0.14 k–u	3.82 ± 0.22 m–r	7.80 ± 0.83 c–h
K125306	VF018	6.61 ± 0.23 g–h	10.61 ± 0.12 d–g	17.22 ± 0.23 f–i	2.82 ± 0.03 i–q	9.47 ± 0.13 a	8.67 ± 0.30 a–d
K125310	VF019	5.20 ± 0.15 k–n	7.87 ± 0.16 v–z	13.07 ± 0.28 o–u	2.98 ± 0.06 g–m	5.74 ± 0.08 d–f	8.89 ± 0.23 a–c
K125311	VF020	4.89 ± 0.24 m–o	6.70 ± 0.48 af	11.59 ± 0.71 w–z	2.57 ± 0.02 k–v	6.67 ± 0.32 b,c	7.52 ± 1.08 c–k
K125312	VF021	4.69 ± 0.18 n–p	7.43 ± 0.18 z–ac	12.12 ± 0.32 u–x	3.07 ± 0.03 f–j	5.60 ± 0.31 d–g	6.95 ± 0.47 e–o
K125315	VF022	5.25 ± 0.12 k–n	8.33 ± 0.08 r–v	13.58 ± 0.17 n–q	3.23 ± 0.08 e–i	3.82 ± 0.16 m–r	7.94 ± 0.94 c–g
K125318	VF023	3.20 ± 0.11 s–x	6.96 ± 0.09 ac–af	10.16 ± 0.19 aa–ab	2.40 ± 0.09 p–v	4.22 ± 0.19 k–p	7.75 ± 0.79 c–i
K125321	VF024	7.59 ± 0.83 e–f	10.06 ± 0.88 h–k	17.65 ± 1.70 d–i	3.03 ± 0.33 g–k	5.90 ± 0.78 d,e	9.41 ± 0.91 a,b
K125323	VF025	3.67 ± 1.36 q–v	7.76 ± 0.22 x–aa	11.43 ± 1.53 x–z	3.58 ± 0.15 c–e	7.22 ± 0.30 b	8.03 ± 0.36 c–f
K125324	VF026	3.87 ± 0.08 q–u	9.43 ± 0.37 m–p	13.30 ± 0.41 n–t	2.33 ± 0.14 r–w	3.88 ± 0.40 m–r	7.07 ± 0.47 e–n
K125325	VF027	3.15 ± 0.15 t–x	9.00 ± 0.15 p–q	12.15 ± 0.29 u–x	1.74 ± 0.07 y–z	3.47 ± 0.22 p–u	7.79 ± 0.28 c–h
K125326	VF028	9.74 ± 0.75 a	9.60 ± 0.39 k–o	19.34 ± 1.12 a	1.91 ± 0.22 w–z	4.34 ± 0.41 j–o	7.13 ± 0.79 e–n
K125329	VF029	8.61 ± 0.26 c,d	10.35 ± 0.13 f–i	18.96 ± 0.34 a–c	2.76 ± 0.22 j–s	5.85 ± 0.53 d,e	7.11 ± 0.16 e–n
K151258	VF030	3.46 ± 0.07 r–w	10.90 ± 0.32 d–e	14.36 ± 0.30 L–n	2.97 ± 0.36 g–n	4.44 ± 0.48 i–n	7.14 ± 0.99 e–n
K152838	VF031	6.53 ± 0.38 h,i	9.57 ± 0.26 k–o	16.10 ± 0.63 j–k	1.87 ± 0.29 x–z	4.99 ± 0.57 f–k	6.84 ± 0.92 e–p
K152839	VF032	5.21 ± 0.10 k–n	8.33 ± 0.14 r–v	13.54 ± 0.06 n–r	1.65 ± 0.04 z	5.70 ± 0.46 d–g	5.72 ± 0.74 m–v
K173330	VF033	4.36 ± 0.08 o–q	8.61 ± 0.55 q–u	12.97 ± 0.63 p–u	3.09 ± 0.22 f–j	4.37 ± 0.40 j–o	5.45 ± 0.81 p–x
K173331	VF034	8.80 ± 0.28 b–d	10.52 ± 0.01 e–h	19.32 ± 0.28 a	3.34 ± 0.18 d–g	3.16 ± 0.42 r–x	5.11 ± 0.64 s–x
K173333	VF035	8.49 ± 0.34 c,d	10.08 ± 0.08 h–k	18.57 ± 0.39 a–d	4.08 ± 0.10 a,b	3.65 ± 0.19 n–s	5.57 ± 0.76 o–w
K173335	VF036	9.65 ± 0.48 a	8.36 ± 0.18 r–v	18.01 ± 0.56 c–h	4.38 ± 0.42 a	3.66 ± 0.58 n–s	5.44 ± 0.18 p–x
K193529	VF037	9.13 ± 0.40 a–c	10.01 ± 0.39 i–l	19.14 ± 0.69 a,b	3.41 ± 0.18 d–g	3.27 ± 0.34 q–w	6.15 ± 0.24 k–t
K193532	VF038	9.13 ± 0.32 a–c	8.26 ± 0.15 t–x	17.39 ± 0.34 e–i	3.84 ± 0.28 b,c	3.20 ± 0.49 r–x	6.15 ± 0.14 k–t
K193535	VF039	7.22 ± 0.52 f–g	10.71 ± 0.51 d–g	17.92 ± 1.02 c–h	2.85 ± 0.06 h–p	2.78 ± 0.20 t–aa	5.11 ± 0.13 s–x
K193541	VF040	8.13 ± 0.44 d,e	3.41 ± 0.19 ag	11.55 ± 0.63 w–z	3.29 ± 0.35 e–h	3.48 ± 0.43 p–u	4.91 ± 0.14 t–x
K193648	VF041	3.52 ± 0.09 r–w	10.62 ± 0.12 d–g	14.14 ± 0.03 m–o	3.01 ± 0.15 g–l	2.61 ± 0.11 v–aa	5.37 ± 0.18 q–x
K193657	VF042	5.22 ± 0.20 k–n	8.48 ± 0.04 r–u	13.70 ± 0.24 n–q	3.27 ± 0.31 e–h	3.60 ± 0.53 o–t	5.71 ± 0.85 n–v
K195293	VF043	2.86 ± 0.12 w–y	9.74 ± 0.16 j–o	12.61 ± 0.27 q–w	3.48 ± 0.12 c–f	2.75 ± 0.21 u–aa	6.77 ± 0.13 e–q
K195298	VF044	2.23 ± 0.06 y–aa	11.46 ± 0.13 b,c	13.69 ± 0.15 n–q	3.38 ± 0.39 d–g	3.39 ± 0.25 p–v	5.83 ± 0.35 L–u
K195301	VF045	3.30 ± 0.54 s–x	9.51 ± 0.35 L–o	12.81 ± 0.20 q–v	3.22 ± 0.02 e–i	3.05 ± 0.20 r–x	7.24 ± 0.86 e–l
K195304	VF046	3.48 ± 0.23 r–w	7.91 ± 0.22 v–z	11.39 ± 0.44 x–z	2.58 ± 0.17 k–v	3.31 ± 0.12 q–w	7.11 ± 0.11 e–n
K195305	VF047	3.25 ± 0.24 s–x	10.19 ± 0.39 g–j	13.44 ± 0.62 n–s	1.88 ± 0.12 x–z	2.62 ± 0.14 v–aa	4.23 ± 0.78 w–x
K195311	VF048	2.61 ± 0.10 x–z	9.77 ± 0.39 j–o	12.38 ± 0.48 s–x	3.30 ± 0.59 e–h	3.26 ± 0.37 r–w	7.39 ± 1.35 d–k
K195819	VF049	2.18 ± 0.07 y–aa	7.33 ± 0.12 aa–ad	9.51 ± 0.14 ab	2.83 ± 0.14 i–q	3.70 ± 0.27 n–s	5.46 ± 0.47 p–x
K195821	VF050	1.96 ± 0.29 z–aa	7.23 ± 0.22 ab–ae	9.19 ± 0.28 ab	1.96 ± 0.10 w–z	1.70 ± 0.17 ab	4.34 ± 0.47 v–x
K195855	VF051	3.11 ± 0.05 v–x	8.28 ± 0.21 t–w	11.39 ± 0.17 x–z	3.65 ± 0.12 c–e	3.30 ± 0.28 q–w	7.04 ± 0.33 e–n
K204279	VF052	7.41 ± 0.04 f	7.46 ± 0.24 z–ac	14.86 ± 0.20 L–m	2.50 ± 0.05 o–v	1.70 ± 0.12 ab	6.27 ± 0.40 j–t
K204869	VF053	3.58 ± 0.10 r–w	9.34 ± 0.07 o–p	12.92 ± 0.04 q–u	3.33 ± 0.01 d–g	4.15 ± 0.09 L–p	9.48 ± 0.64 a
K204870	VF054	3.90 ± 0.09 q–s	7.86 ± 0.26 v–z	11.76 ± 0.29 v–y	3.24 ± 0.21 e–i	5.27 ± 0.54 e–h	8.19 ± 0.10 b–e
K204874	VF055	6.13 ± 0.16 h–j	11.60 ± 0.09 b	17.72 ± 0.20 d–i	2.76 ± 0.15 j–r	2.05 ± 0.15 aa–ab	7.34 ± 0.18 d–k
K204875	VF056	8.86 ± 0.20 b,c	9.58 ± 0.33 k–o	18.44 ± 0.46 a–e	2.53 ± 0.19 n–v	2.55 ± 0.24 w–aa	7.88 ± 0.59 c–h
K204878	VF057	5.12 ± 0.33 L–n	7.50 ± 0.29 z–ab	12.62 ± 0.57 q–w	2.86 ± 0.22 h–o	2.02 ± 0.26 aa–ab	9.50 ± 0.65 a
K204879	VF058	5.89 ± 0.09 i–k	11.08 ± 0.22 c,d	16.97 ± 0.15 h–j	2.56 ± 0.05 L–v	2.43 ± 0.18 x–ab	7.11 ± 0.46 e–n
K204881	VF059	5.33 ± 0.18 k–n	8.13 ± 0.03 u–y	13.46 ± 0.20 n–s	2.42 ± 0.12 o–v	2.17 ± 0.15 y–ab	6.68 ± 0.09 f–r
K204882	VF060	4.68 ± 0.07 n–p	10.55 ± 0.27 e–h	15.23 ± 0.20 k–l	2.50 ± 0.04 o–v	2.93 ± 0.14 s–y	7.32 ± 0.22 d–k
K260904	VF061	3.32 ± 0.09 s–x	6.78 ± 0.05 ae–af	10.10 ± 0.05 aa–ab	2.47 ± 0.26 o–v	3.39 ± 0.37 p–v	8.06 ± 0.76 c–f
K260906	VF062	3.33 ± 0.25 s–x	9.77 ± 0.34 j–o	13.09 ± 0.58 o–u	2.14 ± 0.03 v–y	3.12 ± 0.09 r–x	6.48 ± 0.53 h–s
K260907	VF063	2.63 ± 0.09 x–z	8.81 ± 0.20 q–s	11.45 ± 0.28 x–z	2.39 ± 0.14 q–v	5.23 ± 0.70 e–h	6.97 ± 1.09 e–o
K260908	VF064	3.05 ± 0.14 v–x	9.89 ± 0.24 i–n	12.93 ± 0.35 p–u	2.54 ± 0.26 m–v	3.31 ± 0.51 q–w	6.50 ± 0.33 g–s
K260910	VF065	3.30 ± 0.18 s–x	10.74 ± 0.22 d–f	14.04 ± 0.39 m–p	2.31 ± 0.18 s–w	3.61 ± 0.36 o–t	6.50 ± 0.32 g–s
K260911	VF066	1.75 ± 0.07 aa	9.94 ± 0.49 i–m	11.70 ± 0.55 w–y	1.93 ± 0.05 w–z	2.62 ± 0.09 v–aa	6.77 ± 0.16 e–q
K260912	VF067	2.22 ± 0.12 y–aa	9.39 ± 0.24 n–p	11.61 ± 0.34 w–z	2.20 ± 0.04 u–x	2.62 ± 0.13 v–aa	6.31 ± 0.05 i–t
K261723	VF068	6.55 ± 0.26 h,i	11.53 ± 0.23 b,c	18.08 ± 0.34 c–g	2.51 ± 0.10 o–v	2.11 ± 0.13 z–ab	6.94 ± 0.17 e–o

*Note:* Different unpaired (a–z) or paired (aa–az) letters indicate significantly different mean values in a column (*p* < 0.05).

### 3.3 Nutritional Components

The variations of all nutritional components across the entire faba bean population are summarized in Table 2, while the contents of each for individual accessions are provided in Table S5. Crude fiber, dietary fiber, total fat, and total protein contents were in the ranges of 3.42% to 10.85%, 9.18% to 25.47%, 0.90% to 1.99%, and 21.68% to 34.40%, respectively, with all components showing significant variation among the accessions (*p* < 0.05). Crude fiber exhibited the highest CV (32.85%), while total protein had the lowest (9.61%). Accessions VF032, VF009, and VF041 showed the highest dietary fiber, crude fiber, and total protein contents, respectively, each of which was significantly different from all other accessions. Except for four accessions, the highest total fat content observed in accession VF065 was significantly higher than that of all other accessions (Table S5). In contrast, accessions VF025, VF035, VF034, and VF008 had the lowest dietary fiber, crude fiber, total fat, and total protein contents, respectively.

**TABLE 2:** Statistical data on the variations of all analyzed biochemical traits across 68 faba bean accessions (total population).

Category	Parameter	Minimum	Maximum	Mean	SD	90th percentile	3rd quartile	Median	1st quartile	10th percentile	CV (%)
Nutritional	Crude fiber (%)	3.42	10.85	6.45	2.12	9.31	8.51	5.85	4.44	3.80	32.85
Components	Dietary fiber (%)	9.18	25.47	15.81	2.77	20.11	17.11	15.81	13.83	12.38	17.54
	Total fat (%)	0.90	1.99	1.33	0.28	1.80	1.46	1.31	1.11	0.95	21.18
	Total protein (%)	21.68	34.40	27.47	2.64	30.95	28.88	27.54	26.05	23.31	9.61
Fatty acids and	Palmitic acid (%)	15.13	18.73	16.76	0.76	17.99	17.25	16.71	16.31	16.30	9.38
Lipid quality	Stearic acid (%)	1.96	3.29	2.57	0.31	3.02	2.73	2.55	2.34	2.36	11.85
	Oleic acid (%)	23.67	40.29	30.57	3.11	34.20	32.47	30.85	28.20	28.53	10.34
	Linoleic acid (%)	38.51	54.93	47.83	2.75	51.85	49.41	47.76	45.86	45.99	6.00
	Linolenic acid (%)	1.58	2.90	2.28	0.27	2.64	2.47	2.23	2.08	2.10	12.27
	TSFA (%)	17.65	21.39	19.33	0.79	20.60	19.83	19.25	18.82	18.92	8.19
	PUFA (%)	78.61	82.35	80.67	0.79	54.10	81.08	80.75	48.17	80.17	5.94
	TUFA (%)	43.72	56.08	50.13	2.36	81.61	51.52	50.42	80.03	48.42	1.98
	DBI	123.84	140.84	133.06	2.88	136.28	134.95	132.99	131.03	131.19	2.77
	TUFA: TSFA	3.68	4.66	4.18	0.21	4.44	4.28	4.20	4.01	4.04	7.56
Antioxidant	DPPH (mg AAE/g)	0.44	2.33	1.05	0.38	1.52	1.28	0.98	0.78	0.61	36.64
Activities	FRAP (mg AAE/g)	0.67	4.94	1.78	0.74	2.45	2.17	1.66	1.35	1.03	41.31
	ABTS (mg TE/g)	1.87	7.26	4.36	1.10	5.85	5.16	4.24	3.79	2.81	25.14

Abbreviations: ABTS, ABTS^•+^ scavenging activity; CV, coefficient of variation; DPPH, DPPH^•^ scavenging activity; FRAP, ferric reducing power; PUFA, polyunsaturated fatty acid; SD, standard deviation; TSFA, total saturated fatty acid; TUFA, total unsaturated fatty acid.

### 3.4 Fatty Acids Contents

This study detected target fatty acids in all faba bean accessions at significantly different concentrations (*p* < 0.05), with CVs of ≥ 6.00%. The contents of individual and total fatty acids for each accession are presented in Figure S1, while the summary statistical data is provided in Table 2. Palmitic acid, the most abundant saturated fatty acid, ranged from 15.13% to 18.73%, whereas linoleic acid, the dominant unsaturated fatty acid, ranged from 38.51% to 54.93%. Stearic acid, oleic acid, and linolenic acid were found in the ranges of 1.96%–3.29%, 23.67%–40.29%, and 1.58%–2.90%, respectively. Accession VF027 exhibited the highest palmitic acid and TSFA, as well as the lowest TUFA, each being significantly different from all other accessions (*p* < 0.05). This accession also had the lowest TUFA‐to‐TSFA ratio. Similarly, accession VF012 showed the highest stearic acid and oleic acid, as well as the lowest linoleic acid, with the latter two being significantly different from all other accessions. The stearic acid content in accession VF012 was also significantly different from all accessions except for accession VF042. Additionally, accession VF012 had the lowest DBI. Accessions VF053 and VF062 had the highest linoleic acid and linolenic acid contents, respectively, each being significantly different from all other accessions. The former also had the highest DBI. In contrast, accession VF063 showed the lowest palmitic acid and TSFA. This accession also had the highest TUFA and TUFA‐to‐TSFA ratio. Accessions VF058, VF065, and VF063 exhibited the lowest stearic acid, oleic acid, and linolenic acid contents, respectively. Overall, PUFA was the most abundant in all accessions, followed by monounsaturated fatty acids (MUFA) and TSFA.

### 3.5 Antioxidant Activities

The antioxidant activities of each faba bean accession were determined using three independent assays, including DPPH^•^ scavenging activity, ABTS^•+^ scavenging activity, and FRAP. The antioxidant activities of each accession are presented in Table S5, while summary statistical data are provided in Table 2. Significant variations in antioxidant activity were observed across all accessions. DPPH^•^ scavenging activity and FRAP were in the ranges of 0.44–2.33 mg AAE/g and 1.87–7.26 mg AAE/g, respectively. Similarly, ABTS^•+^ scavenging activity varied between 0.67 and 4.49 mg TE/g. All parameters exhibited a CV above 25.00%. Accession VF012 had the highest DPPH^•^ scavenging activity and ABTS^•+^ scavenging activity, each being significantly different from all other accessions, except for VF004 and VF009, respectively. Additionally, accession VF012 showed the second‐highest FRAP, following VF004. The FRAP value in accession VF004 was significantly different from all other accessions. In contrast, accessions VF050, VF052, and VF027 exhibited the lowest DPPH^•^ scavenging activity, ABTS^•+^ scavenging activity, and FRAP, respectively. Accession VF052 also had the second‐lowest DPPH^•^ scavenging activity and the fifth‐lowest FRAP, while VF027 had the third‐lowest DPPH^•^ scavenging activity and the sixth‐lowest ABTS^•+^ scavenging activity.

### 3.6 Effect of Seed Color Variation on Biochemical Traits

**FIGURE 3:**
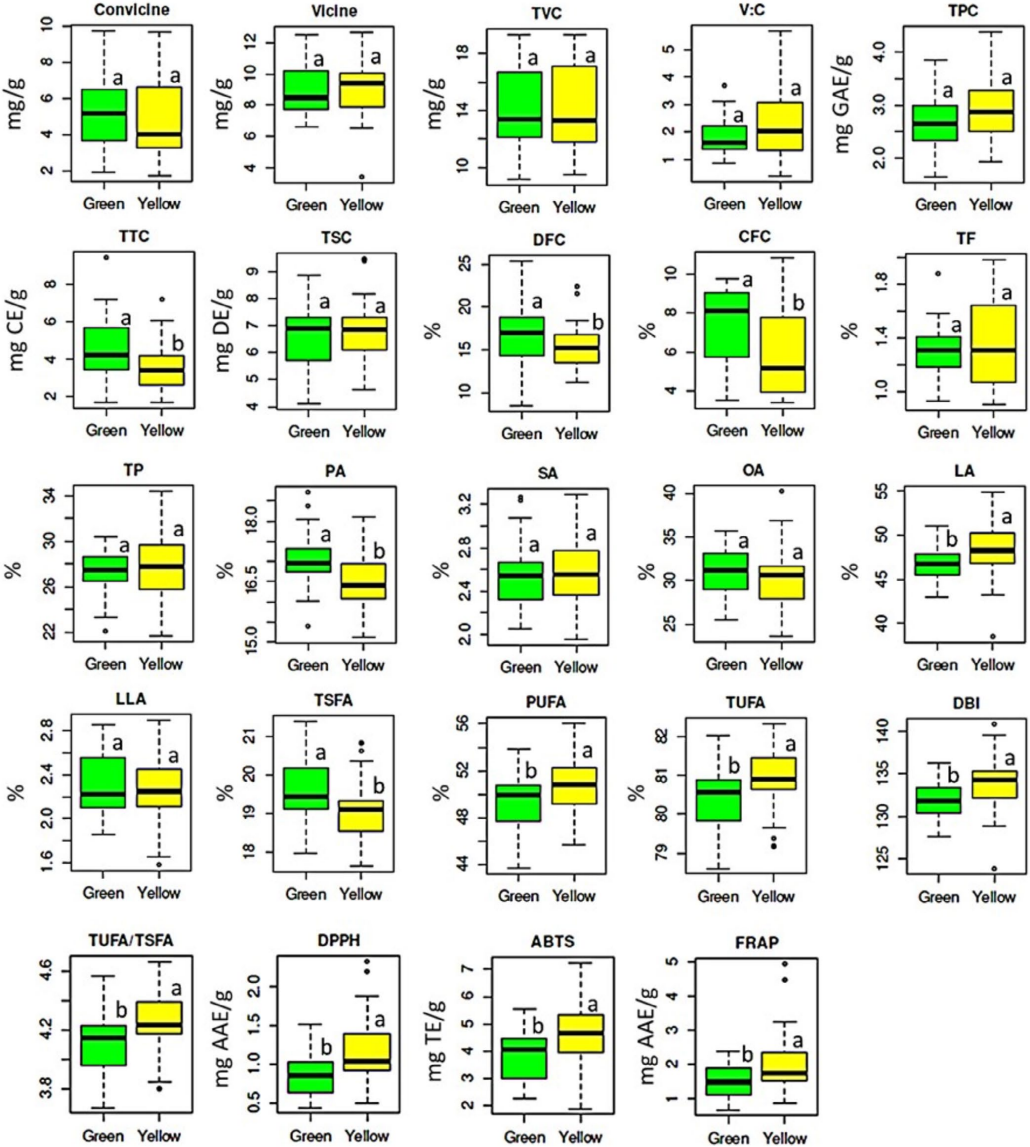
Boxplots showing the variations of all biochemical traits between green and yellow faba bean genotypes. ABTS, ABTS^•+^ scavenging activity; CFC, Crude fiber content; DBI, Double bond index; DFC, Dietary fiber content; DPPH, DPPH^•^ scavenging activity; FRAP, Ferric reducing power; LA, Linoleic acid; LLA, Linolenic acid; OA, Oleic acid; PA, Palmitic acid; PUFA, Polyunsaturated fatty acid; SA, Stearic acid; TF, Total fat; TP, Total protein; TPC, Total phenol content; TSC, Total saponin contents; TSFA, Total saturated fatty acid; TTC, Total tannin content; TUFA, Total unsaturated fatty acid; TVC, Total vicine‐convicine content; V:C, Vicine‐to‐convicine ratio. Different unpaired letters on boxplots represent significantly different means in a category (*p* < 0.05).

The effect of seed color variation on the levels of each biochemical trait was statistically analyzed (Figure 3, Table S6). On average, yellow faba beans had higher vicine content (9.13 mg/g) and vicine‐to‐convicine ratio (2.27), while green faba beans had higher convicine (5.40 mg/g) and total vicine‐convicine (TVC) content (14.20 mg/g). These variations, however, were not statistically significant. Furthermore, yellow faba beans exhibited higher total phenol (2.91 mg GAE/g) and total saponin (6.79 mg DE/g) contents, while green faba beans had a higher total tannin content (4.63 mg CE/g), the latter showing a significant variation (*p* < 0.05). Several nutritional parameters also showed significant variations. On average, green faba beans had significantly higher crude fiber (7.52%) and dietary fiber contents (16.85%) compared to yellow faba beans (5.79% and 15.32%, respectively). In contrast, yellow faba beans had higher total fat (1.35%) and total protein (27.60%) contents. Unlike crude and dietary fiber contents, the variations in total fat and total protein contents were not statistically significant (Figure 3, Table S6). Regarding fatty acids, green faba beans had higher values for all individual fatty acids except for linoleic acid content. Conversely, yellow faba beans exhibited higher TUFA, DBI, and TUFA‐to‐TSFA ratio. Among these, palmitic acid, linoleic acid, total fatty acid contents, DBI, and the TUFA‐to‐TSFA ratio showed significant variations. Seed color variation also significantly affected all antioxidant activities, with yellow faba beans exhibiting higher DPPH^•^ scavenging activity (1.17 mg AAE/g), ABTS^•+^ scavenging activity (4.69 mg TE/g), and FRAP (1.97 mg AAE/g) than green faba beans (Figure 3, Table S6).

### 3.7 Multivariate Analysis

**FIGURE 4:**
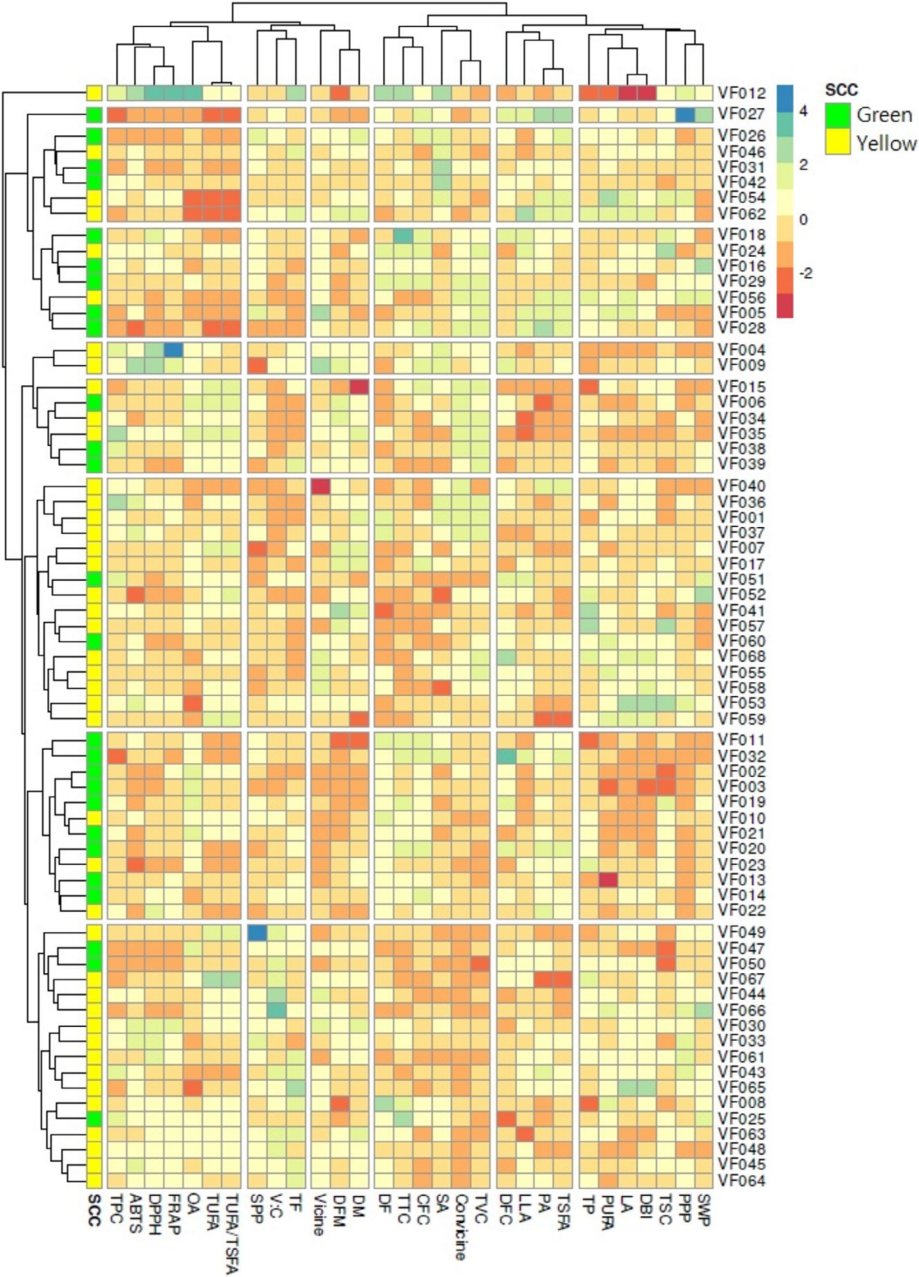
Tow‐way hierarchical clustering heatmap showing the distribution of antinutrient factors, nutritional parameters, and antioxidant activities across 68 faba bean accessions. ABTS, ABTS^•+^ scavenging activity; CFC, Crude fiber content, DBI, Double bond index; DF, Days to flowering; DFC, Dietary fiber content; DFM, Days from flowering to maturity; DM, Days to maturity; DPPH, DPPH^•^ scavenging activity; FRAP, Ferric reducing power; LA, Linoleic acid; LLA, Linolenic acid; OA, Oleic acid; PA, Palmitic acid; PPP, Number of pods per plant; PUFA, Polyunsaturated fatty acid; SA, Stearic acid; SPP, Number of seeds per pod; SWP, Seed weight per plant; TF, Total fat; TP, Total protein; TPC, Total phenol content; TSC, Total saponin contents; TSFA, Total saturated fatty acid; TTC, Total tannin content; TUFA, Total unsaturated fatty acid; TVC, Total vicine‐convicine content; V:C, Vicine‐to‐convicine ratio.

**FIGURE 5:**
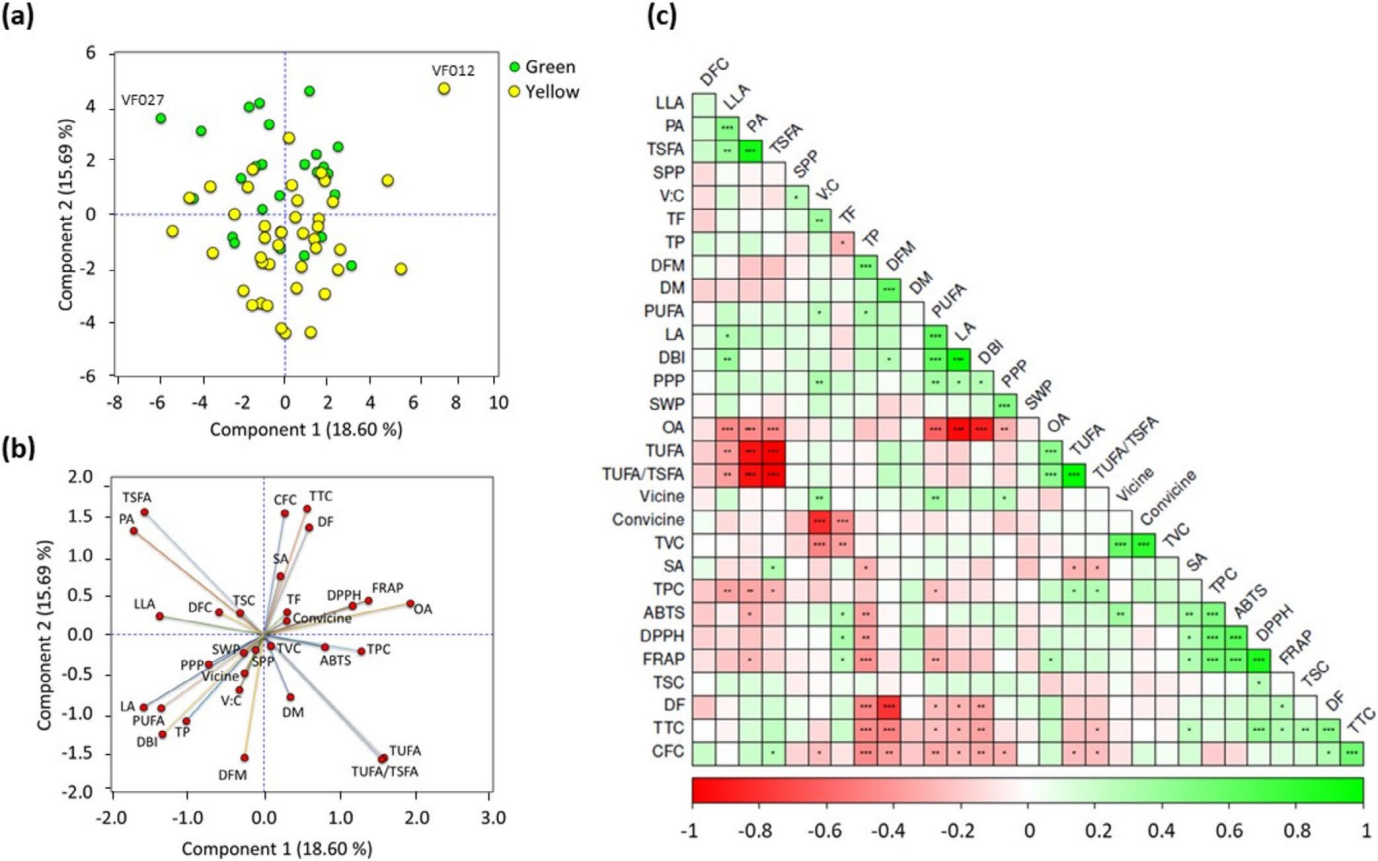
Score plot of faba bean accessions (a), loading plot of all analyzed parameters (b) obtained from principal component analysis, and pair‐wise Pearson's correlation coefficient value (r) between the analyzed parameters and their levels of significance (c). ABTS, ABTS^•+^ scavenging activity; CFC, Crude fiber content, DBI, Double bond index; DF, Days to flowering; DFC, Dietary fiber content; DFM, Days from flowering to maturity; DM, Days to maturity; DPPH, DPPH^•^ scavenging activity; FRAP, Ferric reducing power; LA, Linoleic acid; LLA, Linolenic acid; OA, Oleic acid; PA, Palmitic acid; PPP, Number of pods per plant; PUFA, Polyunsaturated fatty acid; SA, Stearic acid; SPP, Number of seeds per pod; SWP, Seed weight per plant; TF, Total fat; TP, Total protein; TPC, Total phenol content; TSC, Total saponin contents; TSFA, Total saturated fatty acid; TTC, Total tannin content; TUFA, Total unsaturated fatty acid; TVC, Total vicine‐convicine content; V:C, Vicine‐to‐convicine ratio. *p < 0.05, **p < 0.01, ***p < 0.001.

The entire dataset obtained from agronomical traits, nutritional components, antinutrients, fatty acids, and antioxidant capacities was analyzed for HCA, PCA, and correlation analysis. The HCA categorized the faba bean accessions into eight groups (Figure 4). Accessions VF027 and VF012 were separated from the rest of the groups. The largest number of accessions was found in cluster I (*n* = 17) followed by clusters III (*n* = 16) and II (*n* = 12), the former two being dominated by yellow genotypes. Clusters IV and VII had an equal number of accessions (*n* = 6) as well as equal proportions of green and yellow genotypes. The other clusters, including VI and V, had 7 and 2 accessions, respectively, the former being dominated by green genotypes while both being yellow in the latter. All the parameters, except for DM, SPP, and saponin content, showed significant variations between the clusters (Table S7). On average, accessions in cluster V had the highest DM, vicine, dietary fiber, crude fiber, total fat, DPPH^•^ scavenging activity, ABTS^•+^ scavenging activity, and FRAP, while having the lowest average DBI, linoleic acid, total protein, and SPP. In contrast, accessions in cluster II exhibited the lowest average DFM, DM, PPP, vicine, TVC, PUFA, and ABTS^•+^ scavenging activity, while having the highest average DF. Other clusters also demonstrated such characteristic features (Table S7). The PCA was analyzed using the variance observed along the first two principal components (Figure 5). Component 1 accounted for 18.60% of the total variance, while component 2 accounted for 15.69%. The faba bean accessions showed wide distribution in the score plot along these two components (Figure 5a) and the loading plot showed a wide‐ranging association of the accessions with the analyzed parameters (Figure 5b). Interestingly, the score plot based on clusters supported the HCA observation (Figure S2). Total phenol content, oleic acid, linoleic acid, linolenic acid, and FRAP were the major contributors along PC1 and had FL > ±0.50 and contributions of above 6.00% (Figure 5b, Table 3). Likewise, DF, DFM, total tannin, and crude fiber were the major contributors along PC2, with contributions exceeding 7.00%. Palmitic acid, TSFA, TUFA, and DBI had comparable contributions along both components. The association of all parameters observed in the loading plot (Figure 5b) and HCA was further viewed using Pearson's correlation (Figure 5c). Among the agronomic traits, DFM had a strong positive correlation with DM (*r* = 0.61) and a negative correlation with DF (*r* = −0.80), each being significant at *p* < 0.001. Moreover, SWP and PPP showed a strong correlation with each other (*r* = 5.88, *p* < 0.001). On the other hand, oleic acid showed a negative and/or weak correlation with the remaining fatty acids, particularly with the unsaturated fatty acids, including linolenic acid and linoleic acid. TSFA and TUFA also had an inverse relationship with each other (*r* = −0.999. *p* < 0.001). Protein and fat contents also showed a negative correlation with each other (*r* = −0.279, *p* < 0.05). Total phenol had a strong and significant correlation with all antioxidant activities (*r* ≥ 0.488, *p* < 0.001). Total tannin and total saponin contents also exhibited positive correlations with antioxidant activities, but none of the correlations were significant.

**TABLE 3:** Factor loading (FL) and contributions (%) of all analyzed traits to the variances observed along the first two principal components.

Trait	Parameter	Component 1		Component 2	
		FL	%	FL	%
Agronomic	Days to flowering	0.25	1.13	0.58	7.18
	Days from flowering to maturity	−0.11	0.22	−0.67	9.40
	Days to maturity	0.15	0.38	−0.34	2.41
	Number of seeds per pod	−0.05	0.04	−0.08	0.15
	Number of pods per plant	−0.31	1.73	−0.16	0.54
	Seed weight per plant	−0.11	0.24	−0.10	0.20
Antinutrients	Convicine	0.13	0.29	0.07	0.12
	Vicine	−0.11	0.22	−0.21	0.91
	Total vicine‐convicine	0.04	0.02	−0.06	0.08
	Vicine‐to‐convicine ratio	−0.14	0.34	−0.30	1.89
	Total phenol content	0.54	5.28	−0.09	0.17
	Total tannin content	0.24	1.02	0.68	9.89
	Total saponin content	−0.13	0.32	0.12	0.30
Nutritional components	Dietary fiber content	−0.25	1.15	0.12	0.32
	Crude fiber content	0.11	0.24	0.66	9.19
	Total fat content	0.13	0.29	0.12	0.32
	Total protein content	−0.44	3.40	−0.47	4.64
Fatty acids	Palmitic acid	−0.73	9.53	0.56	6.72
	Stearic acid	0.09	0.14	0.32	2.13
	Oleic acid	0.82	11.92	0.17	0.62
	Linoleic acid	−0.67	8.09	−0.39	3.30
	Linolenic acid	−0.58	6.09	0.10	0.22
	Total saturated fatty acid	−0.67	8.00	0.67	9.41
	Polyunsaturated fatty acid	−0.57	5.92	−0.40	3.35
	Total unsaturated fatty acid	0.67	8.01	−0.67	9.40
	Double bond index	−0.57	5.80	−0.54	6.17
	TUFA‐to‐TSFA ratio	0.65	7.69	−0.67	9.62
Antioxidant activity	DPPH scavenging activity	0.49	4.35	0.16	0.53
	ABTS^•+^ scavenging activity	0.34	2.08	−0.07	0.10
	Ferric reducing antioxidant power	0.58	6.08	0.19	0.73
Eigenvalue		5.58		4.71	
Variability (%)		18.60		15.69	
Cumulative (%)		18.60		34.29	

Abbreviations: TSFA, Total saturated fatty acid; TUFA, Total unsaturated fatty acid.

## 4 Discussion

### 4.1 General

In addition to their agro‐morphological traits, a comprehensive analysis of biochemical properties in crop genetic materials is critical to optimize their application in the food industry, breeding programs, and agriculture. This is particularly important for crops like faba beans, which contain both beneficial and undesirable biochemical components (Lippolis et al. 2025; Khazaei et al. 2019). In this study, significant variations were observed in agro‐morphological traits, nutritional components, antinutrients, fatty acids, and antioxidant capacities across 68 faba bean accessions, with a CV ranging from 1.98% to 53.25%. These variations highlight the wide genetic diversity among the accessions, offering a range of options for selecting genotypes with desirable traits for specific applications (Akgun and Canci 2023; Gutierrez et al. 2006).

### 4.2 Agronomic Performances of Faba Bean Accessions

A total of thirteen agro‐morphological parameters were evaluated in this study, revealing wide variation among the faba bean accessions. The distributions observed in categorical traits were consistent with previous reports (Alharbi and Adhikari 2020; Helios et al. 2021; Li and Yang 2014). Among these traits, growth habit and pod shape exhibited the highest variability, whereas branch status and pod angle showed moderate variation. Previous studies revealed that growth habit, branch configuration, and pod morphology are associated with yield potential in faba beans, highlighting the relevance of such diversity in selecting those varieties with desirable qualities (Alharbi and Adhikari 2020; Helios et al. 2021; Li and Yang 2014). Quantitative agronomic traits also displayed wide variation, with yield‐related parameters showing more than six‐fold differences across the accessions. Previous studies also observed wide variation in several quantitative agronomical traits between different faba bean genotypes. For instance, Hiywotu et al. (2022) reported DF ranging from 42 to 59 days and DM from 109 to 131 days among 81 faba bean accessions grown in Ethiopia. In the same study, PPP ranged from 3.5 to 39.2 and SPP varied between 1 and 38. In another study, Ammar et al. (2015) evaluated 40 genotypes grown in Saudi Arabia and reported wide‐ranging values for several traits, including DF (33–78 days), DM 9(112–154 days), PPP (8.8–44.3), and seed yield per plant (14.9–70.8 g). Some of these ranges were comparable to those observed in our study, while others were much wider and/or narrower. Such discrepancies among reported values could be attributed to genotypic differences, environmental factors, cultivation practices, and seasonal variations (Sellami et al. 2021). Early‐maturing and high‐yielding genotypes are particularly valuable in the context of food production, breeding programs, and sustainable agriculture (Akgun and Canci 2023; Alharbi and Adhikari 2020; Yehmed et al. 2022). In this study, 11 accessions exhibited DM below the first quartile (25th percentile). Among these, six accessions (VF015, VF011, VF059, VF005, VF003, and VF019) had DM below the 10th percentile, indicating early maturity. Notably, accession VF019 demonstrated both SPP and SWP above the third quartile (75th percentile). Interestingly, accession VF027 showed PPP and SWP above the 90th percentile. These findings suggest that both VF019 and VF027 possess superior agronomic performance (Table S8), and may serve as promising candidates for use in agriculture and breeding programs (Akgun and Canci 2023; Alharbi and Adhikari 2020; Khazaei et al. 2021).

### 4.3 Diversity of Antinutrient Factors

Antinutrient factors are a major concern in legumes due to their negative effects on nutrient digestibility, bioavailability, and overall food quality (De Silva et al. 2024; Khazaei et al. 2019). These compounds can also limit the industrial utilization of legume crops and hence, understanding their distribution and concentration is crucial. In this study, key antinutrient components in faba beans, such as vicine, convicine, TVC, total phenols, total tannins, and total saponins, showed significant variation among the accessions, with differences exceeding two‐fold. In a more recent study, vicine content ranged from 3.48 to 10.02 g/kg across 189 Indian faba bean accessions (Debnath et al. 2025). Similarly, vicine and convicine levels in five Canadian cultivars ranged from 0.40 to 3.93 mg/g and 1.02 to 9.94 mg/g, respectively (Wei et al. 2022). Tannin content has been reported to vary from 0.00 to 12.5 mg CE/g, total phenols from 1.40 to 5.00 mg GAE/g, and saponins from 18.3 to 109.0 SI/g in different studies (Mayer Labba et al. 2021; Walter et al. 2023). Other studies have also confirmed the broad variability in the levels of these antinutrient factors across different faba bean genotypes (De Silva et al. 2024; Khamassi et al. 2013; Millar et al. 2019; Shi et al. 2022, 2024; Wei et al. 2022). The wide range of values observed may, once again, be attributed to genetic variances and environmental effects (Hefni et al. 2015; Khazaei et al. 2019; Shi et al. 2024; Walter et al. 2023; Westling et al. 2024). Such differences may also result from variations in post‐harvest handling, extraction methods, and analysis protocols (Mazi and Caglayan 2025; Jha et al. 2024; Xu and Chang 2007). Given the unfavorable effects of antinutrient levels, the identification and use of low‐antinutrient genotypes are important for enhancing the overall utilization of faba beans (Singh et al. 2023). In this study, three accessions, including VF066, VF050, and VF040, were identified as having a convicine content below 2.00 mg/g and a vicine content below 4.00 mg/g. Interestingly, these values were lower than the 10th percentile. Additionally, accessions VF050 and VF049 recorded TVC below 10.00 mg/g, while VF040 exhibited the lowest vicine‐to‐convicine ratio. Notably, accession VF050 also displayed much lower tannin (< 2.00 mg CE/g) and saponin (< 4.50 mg DE/g) levels (Table S8). These characteristics make VF050 a good candidate for use in food processing and in the development of improved faba bean varieties with reduced antinutrient content (Khazaei et al. 2019; Gutierrez et al. 2006).

### 4.4 Diversity of Nutritional Components and Fatty Acids

Although faba beans contain anti‐nutritional compounds, global interest in their use has grown in recent years due to their high nutritional value (Dhull et al. 2022; Feng et al. 2024; Meng et al. 2021). In this study, the studied accessions showed significant variation in crude fiber, dietary fiber, total fat, total protein, and fatty acid contents. Crude fiber and dietary fiber, in particular, showed nearly three‐fold differences. Previous studies have reported total fat content ranging from 1.18% to 1.26% and dietary fiber from 11.37% to 16.59% (Mayer Labba et al. 2021; Wei et al. 2022). Similar studies exhibited total protein content ranging from 31.80% to 33.30% (Mayer Labba et al. 2021) and 22.70% to 28.30% (Wei et al. 2022) in untreated faba beans. These values were largely in agreement with those found in most of the accessions analyzed in this study. However, some other studies have reported either broader or narrower ranges, possibly due to genetic variation or environmental effects (Dhull et al. 2022; Meng et al. 2021; Millar et al. 2019; Walter et al. 2023; Yilmaz and Yilmaz 2025). Legume proteins and fibers are of particular interest due to their well‐documented health benefits as highlighted before (Dhull et al. 2022; Meng et al. 2021). Such properties make accessions with elevated levels of these nutrients especially valuable (Hamed et al. 2024; Shi et al. 2024; Thomsen et al. 2025). In this study, accessions VF032, VF009, and VF018 exhibited high dietary fiber (> 21.00%) and crude fiber (> 9.00%) contents simultaneously, while VF041 and VF057 showed a total protein content exceeding 33.00%, all ranking above the 90th percentile (Table 2). These observations highlight the potential of these accessions as nutrient‐rich genetic resources. While protein and fiber have received significant attention, fatty acids in faba beans have been less extensively studied (Sipeniece et al. 2021; Lippolis et al. 2024). Nonetheless, the findings of this study revealed the presence of fatty acids with different degrees of unsaturation. Across all accessions, polyunsaturated fatty acids were the most abundant, followed by monounsaturated and saturated fatty acids. All these observations were consistent with previous reports (Caprioli et al. 2016; De Angelis et al. 2021; Lippolis et al. 2024). There is increasing interest in the use of omega‐6 and omega‐3 fatty acids in the food industry, particularly as dietary supplements. In this context, the significant variation observed in the essential fatty acids, including linoleic and linolenic acids, is especially important. Among all accessions, VF062 showed levels of both linoleic and linolenic acids above the 90th percentile, indicating its potential as a valuable genetic resource. However, a high degree of unsaturation negatively affects oil stability and shelf life due to susceptibility to oxidation (Chen and Liu 2020; Caprioli et al. 2016; Lippolis et al. 2024). In this regard, VF027 recorded the lowest TUFA‐to‐TSFA ratio, while VF012, which had the highest levels of stearic acid and oleic acid, exhibited the lowest DBI (Table S8). These two accessions may serve as sources of oil with improved oxidative stability (Chen and Liu 2020; Tilami and Kouřimská 2022).

### 4.5 Diversity of Antioxidant Activities

Faba beans exhibit several biological activities owing to their diverse and bioactive biochemical components (Mekky et al. 2020; De Angelis et al. 2021). Like other legumes, they are rich in natural antioxidants, making them valuable for health‐oriented food products and functional food development (Singh et al. 2017). In this study, significant variation was observed in DPPH^•^ scavenging activity, ABTS^•+^ scavenging activity, and FRAP among the faba bean accessions, each showing more than threefold differences. Earlier studies also reported wide‐ranging antioxidant activities in different faba bean genotypes (Feng et al. 2024; Orita et al. 2019; Singh et al. 2017). As with other secondary metabolites, antioxidant activity in faba beans is influenced by several factors, including genetic background, environmental conditions, postharvest handling, and analytical procedures such as extraction and assay methods (Xu and Chang 2007). These factors likely explain the variability seen across different studies (Vilariño et al. 2009; Salvador‐Reyes et al. 2024). Among the accessions evaluated, VF012 simultaneously showed high antioxidant activity in all three assays, each exceeding the 90th percentile. Similarly, VF004 displayed strong performance in both DPPH^•^ scavenging and FRAP activities. These accessions may serve as valuable sources of antioxidant compounds for food and health‐related applications (Table S8). Moreover, their high total phenolic content further supports the significant contribution of polyphenols to faba bean antioxidant capacity (Feng et al. 2024; Orita et al. 2019; Singh et al. 2017; Yehmed et al. 2024).

### 4.6 Effect of Seed Color Variation

Seed color in legumes is regulated by several genes and it has been linked to variations in biochemical composition across different species (Akgun and Canci 2023; Gutierrez and Torres 2019; Göl et al. 2017; Wei et al. 2022). However, research specifically examining how seed coat color affects biochemical levels in faba bean is limited (Gutierrez et al. 2006; Mayer Labba et al. 2021). This study found that seed color differences can influence various biochemical traits in faba beans. Among antinutrient factors, tannin content showed significant variation between green and yellow genotypes. Briefly, yellow‐seeded faba beans may be preferable if a lower tannin content is desired compared to green seed genotypes. The variation in tannin level may be related to its higher concentration in the seed coat compared to other antinutrients (Gutierrez and Torres 2019; Singh et al. 2023). Conversely, the results of this study indicate that vicine, convicine, and saponin levels cannot be reliably predicted based on seed color alone. Therefore, genotypes should be evaluated individually. Significant differences were observed in crude fiber, dietary fiber, palmitic acid, and linoleic acid among nutritional components and individual fatty acids. All, except linoleic acid, were higher in green faba beans. The variation in palmitic and linoleic acids, which are the most abundant fatty acids, also contributed to significant differences in total fatty acid content, the TUFA‐to‐TSFA ratio, and the DBI. Accordingly, yellow faba beans may be better sources of omega‐6 fatty acids, while green types could produce oils with improved oxidative stability. For antioxidant activity, yellow faba beans demonstrated significantly higher values across all assays, suggesting their potential as rich sources of antioxidants. Although the effect of seed color on biochemical traits in faba beans remains underexplored, studies in other legumes, such as soybeans, support a link between specific seed colors and increased antioxidant levels (Li et al. 2024; Marathe et al. 2011; Orita et al. 2019; Singh et al. 2017). Overall, these findings suggest that seed coat color may serve as an indirect indicator for certain biochemical traits, including tannin content, fiber levels, palmitic and linoleic acid concentrations, and antioxidant potential in faba beans (Choi et al. 2024; Mekky et al. 2020). Further research involving genomics and metabolomics is necessary to better understand the genetic and metabolic basis of seed color and its relationship with nutritional and functional properties (Zhao et al. 2023).

### 4.7 Multivariate Analysis

To further explore the diversity among the evaluated faba bean accessions, multivariate analyses were conducted using the full‐scaled dataset. Both HCA and PCA differentiated the accessions based on their agro‐morphological and biochemical traits. Accessions VF027 and VF012 were distinctly separated in both analyses, emphasizing their unique profiles compared to the other accessions. Accession VF027 exhibited the highest PPP, SWP, palmitic acid, and TSFA. Conversely, it recorded the lowest TUFA, TUFA‐to‐TSFA ratio, and FRAP, along with the third‐lowest DPPH^•^ scavenging activity, second‐lowest total phenol content, and sixth‐lowest ABTS^•+^ scavenging activity. In contrast, accession VF012 showed the longest DF, the highest levels of stearic acid, oleic acid, and antioxidant activities, and the second‐highest total fat content. It also had the third‐highest total tannin content and the lowest DBI. Despite its strong antioxidant profile and lipid quality, its high tannin and vicine levels decrease the overall performance of accession VF012 as a candidate for the food industry, although it could be used in a targeted breeding program (Caprioli et al. 2016; Chen and Liu 2020; Sipeniece et al. 2021). Among the other clusters, all traits except for DM, SPP, and total saponin content showed significant variations, indicating that PCA and HCA primarily grouped accessions based on overall performance patterns. Correlation analysis further identified several significant relationships among the measured traits. For example, the positive correlation between SWP and PPP indicated higher seed yield for accessions producing a large number of pods (Rani et al. 2023). The negative correlation between total fat and total protein contents may reflect differences in enzyme activities involved in metabolic pathways (Gutierrez et al. 2006; Khamassi et al. 2013; Khazaei et al. 2019). Similarly, the inverse association between oleic acid and other unsaturated fatty acids suggests the regulation of these fatty acids by desaturase enzymes (Caprioli et al. 2016; Chen and Liu 2020). Moreover, strong positive correlations between total phenol content and antioxidant activity confirm the key role of phenolic compounds in antioxidant capacity, despite their antinutrient effects. The consistent positive correlations observed between all antioxidant activities (*r* ≥ 0.56) agree with previous studies (Marathe et al. 2011; Mekky et al. 2020). Overall, the multivariate analyses results provided a comprehensive understanding of trait relationships, reinforcing individual trait evaluation results and identifying accessions with distinct and valuable attributes.

## 5 Conclusion

This study provides a comprehensive overview of the diversity of key agronomic traits and several classes of biochemical components in faba bean genetic materials. The results showed significant variations in growth and yield‐related traits, nutritional components, antinutrients, lipid quality, and antioxidant activities among the faba bean accessions. Superior accessions with early maturity and high‐yielding properties, high levels of antioxidants and nutrients, and lower concentrations of antinutrients were identified. These accessions could potentially be used in the food industry, breeding programs, and farming. Moreover, seed color variation showed a significant effect on several biochemical traits, including tannin content, fiber levels, the most abundant fatty acids, lipid quality parameters, and antioxidant activity. These observations suggest that seed color could serve as a useful marker for classifying faba beans based on these characteristics. Overall, the observed biochemical diversity could provide valuable insights into enhancing the nutritional quality and functionality of faba beans. Moreover, the findings of this study could offer important directions for future genomics and metabolomics studies, supporting the development of faba bean varieties with improved qualities for food production and agricultural applications.

## Author Contributions

**Kebede Taye Desta:** formal analysis (equal), investigation (equal), methodology (equal), software (equal), writing – original draft (equal), writing – review and editing (lead). **Myoung‐Jae Shin:** data curation (equal), formal analysis (equal), resources (equal), supervision (equal). **Sukyeung Lee:** conceptualization (equal), formal analysis (equal), project administration (equal), visualization (equal). **Hyemyeong Yoon:** conceptualization (equal), data curation (equal), investigation (equal), methodology (equal), writing – original draft (equal). **Jungyoon Yi:** formal analysis (equal), investigation (equal), project administration (equal), visualization (equal). **Heon‐Woong Kim:** formal analysis (equal), investigation (equal), resources (equal), software (equal), writing – original draft (equal). **Yu‐Mi Choi:** conceptualization (equal), formal analysis (equal), funding acquisition (lead), methodology (equal), project administration (equal), visualization (equal), writing – original draft (equal), writing – review and editing (equal).

## Ethics Statement

The authors have nothing to report.

## Conflicts of Interest

The authors declare no conflicts of interest.

## Supporting information


**Data S1:** fsn370931‐sup‐0001‐Supinfo.docx.

## Data Availability

All the data related to this study are incorporated in the manuscript and Supporting Information. Further inquiries can be directed to the corresponding author.
