# Supplementary material for: Comprehensive Assessment of Biochemical Traits in Diverse Faba Bean (Vicia faba L.) Germplasm for Selection of Superior Accessions
Source: Food Sci Nutr. 2025 Sep 8;13(9):e70931. doi: 10.1002/fsn3.70931 (PMC12417325; doi:10.1002/fsn3.70931)
Supplement: Supplementary file 1 — Data S1: fsn370931‐sup‐0001‐Supinfo.docx. [file FSN3-13-e70931-s001.docx]

***Supplementary Material***

Comprehensive assessment of biochemical traits in diverse faba bean (*Vicia faba* L.) germplasm for selection of superior accessions

Kebede Taye Desta^a^, Myoung-Jae Shin^a^, Sukyeung Lee^b^, Hyemyeong Yoon^a^, Jungyoon Yi^a^, Heon-Woong Kim^c^, and Yu-Mi Choi^a,*^

^a^National Agrobiodiversity Center, National Institute of Agricultural Sciences, Rural Development Administration, Jeonju 54874, Republic of Korea.

^b^International Technology Cooperation Center, Technology Cooperation Bureau, Rural Development Administration, Jeonju 54875, Republic of Korea.

^c^Department of Agrofood Resources, Food and Nutrition Division, National Institute of Crop and Food Science, Rural Development Administration, Wanju-gun 55365, Republic of Korea.

*****Correspondence: [cym0421@korea.kr](mailto:cym0421@korea.kr) (Y.-M.C)


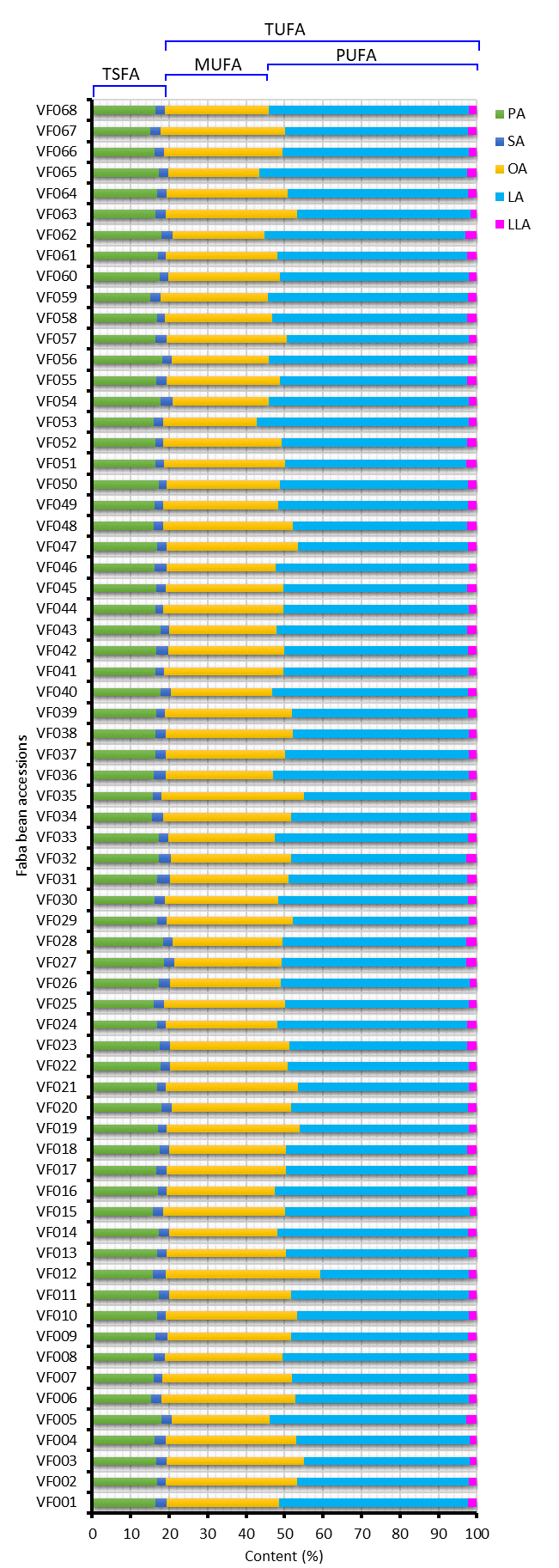


**Figure S1.** Variations of individual and total fatty acid contents across 68 faba bean accessions. PA; Palmitic acid, SA: Stearic acid, OA: Oleic acid, LLA: Linolenic acid, LA: Linoleic acid, TSFA: Total saturated fatty acid, TUFA: Total unsaturated fatty acid, PUFA: Total polyunsaturated fatty acid, MUFA: Monounsaturated fatty acid.


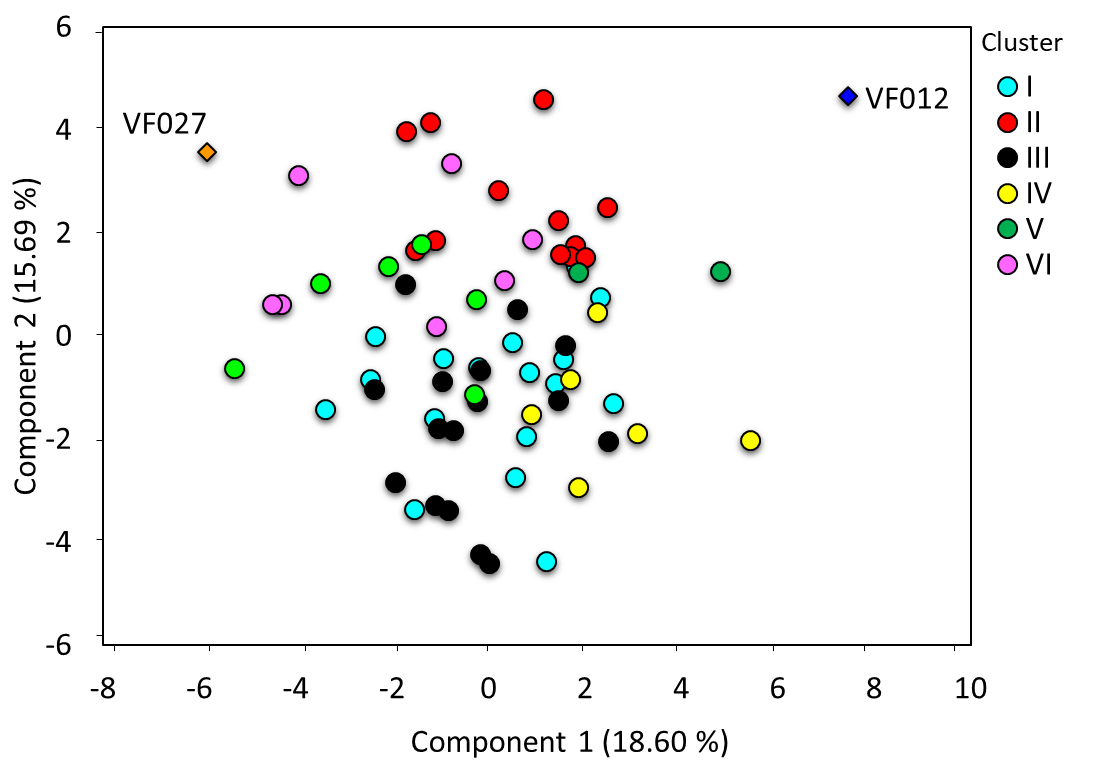


**Figure S2**. Score plot of faba bean accessions based on their grouping as obtained from the HCA.

**Table S1.** List of 68 faba bean accessions investigated in this study, their introduction /temporary numbers as registered at the National Agrobiodiversity Center (Jeonju, Republic of Korea), codes given, and seed color.

| Introduction/  Temporary Number | Code | Seed color |  | Introduction/  Temporary Number | Code | Seed color |
| --- | --- | --- | --- | --- | --- | --- |
| IT-138252 | VF001 | Yellow |  | K173333 | VF035 | Yellow |
| IT-161022 | VF002 | Green |  | K173335 | VF036 | Yellow |
| IT-163348 | VF003 | Green |  | K193529 | VF037 | Yellow |
| IT-188090 | VF004 | Yellow |  | K193532 | VF038 | Green |
| IT-200130 | VF005 | Green |  | K193535 | VF039 | Green |
| IT-203426 | VF006 | Green |  | K193541 | VF040 | Yellow |
| IT-203427 | VF007 | Yellow |  | K193648 | VF041 | Yellow |
| IT-208231 | VF008 | Yellow |  | K193657 | VF042 | Green |
| IT-208235 | VF009 | Yellow |  | K195293 | VF043 | Yellow |
| IT-208722 | VF010 | Yellow |  | K195298 | VF044 | Yellow |
| IT-208723 | VF011 | Green |  | K195301 | VF045 | Yellow |
| IT-208724 | VF012 | Yellow |  | K195304 | VF046 | Yellow |
| IT-208728 | VF013 | Green |  | K195305 | VF047 | Green |
| IT-212781 | VF014 | Green |  | K195311 | VF048 | Yellow |
| IT-228951 | VF015 | Yellow |  | K195819 | VF049 | Yellow |
| K013509 | VF016 | Green |  | K195821 | VF050 | Green |
| K014965 | VF017 | Yellow |  | K195855 | VF051 | Green |
| K125306 | VF018 | Green |  | K204279 | VF052 | Yellow |
| K125310 | VF019 | Green |  | K204869 | VF053 | Yellow |
| K125311 | VF020 | Green |  | K204870 | VF054 | Yellow |
| K125312 | VF021 | Green |  | K204874 | VF055 | Yellow |
| K125315 | VF022 | Yellow |  | K204875 | VF056 | Yellow |
| K125318 | VF023 | Yellow |  | K204878 | VF057 | Yellow |
| K125321 | VF024 | Yellow |  | K204879 | VF058 | Yellow |
| K125323 | VF025 | Green |  | K204881 | VF059 | Yellow |
| K125324 | VF026 | Green |  | K204882 | VF060 | Green |
| K125325 | VF027 | Green |  | K260904 | VF061 | Yellow |
| K125326 | VF028 | Green |  | K260906 | VF062 | Yellow |
| K125329 | VF029 | Green |  | K260907 | VF063 | Yellow |
| K151258 | VF030 | Yellow |  | K260908 | VF064 | Yellow |
| K152838 | VF031 | Green |  | K260910 | VF065 | Yellow |
| K152839 | VF032 | Green |  | K260911 | VF066 | Yellow |
| K173330 | VF033 | Yellow |  | K260912 | VF067 | Yellow |
| K173331 | VF034 | Yellow |  | K261723 | VF068 | Yellow |

**Table S2.** Frequency and relative frequency distributions of categorical agronomic traits across 68 faba bean accessions.

| Trait | Status | *f* | % | H' |
| --- | --- | --- | --- | --- |
| Flower color | White | 62.00 | 91.18 | 0.46 |
|  | Pink | 2.00 | 2.94 |  |
|  | Purple | 4.00 | 5.88 |  |
| Growth habit | Determinate | 35.00 | 51.47 | 0.60 |
|  | Intermediate | 24.00 | 35.29 |  |
|  | Indeterminate | 4.00 | 5.88 |  |
|  | Mixed | 5.00 | 7.35 |  |
| Branch status | Unbranched | 52.00 | 76.47 | 0.50 |
|  | Branched | 0.00 | 0.00 |  |
|  | Mixed | 16.00 | 23.53 |  |
| Pod shape | SCD | 25.00 | 36.76 | 0.60 |
|  | FCM | 30.00 | 44.12 |  |
|  | FNCM | 13.00 | 19.12 |  |
| Pod angle | Drooping | 18.00 | 26.47 | 0.52 |
|  | Erect | 49.00 | 72.06 |  |
|  | Mixed | 1.00 | 1.47 |  |
| Hilum color | Black | 65.00 | 95.59 | 0.43 |
|  | Colorless | 3.00 | 4.41 |  |
| Seed shape | Flat | 63.00 | 92.65 | 0.46 |
|  | Mixed | 2.00 | 2.94 |  |
|  | Round | 2.00 | 2.94 |  |
|  | Angular | 1.00 | 1.47 |  |

*f*: Frequency, FCM: Fat concave in the middle; FNCM: Flat not concave in the middle; H': Standardized Shannon-Weaver index; SCD: Semicylindrical; %: Relative frequency.

**Table S3.** Statistical data on the variations of quantitative agronomical traits across 68 faba bean accessions

| Values | Days to  flowering (days) | Days from flowering  to maturity (days) | Days to maturity  (days) | Number of seeds  per pod (*n*) | Number of pods  per plant (*n*) | Seed weight  per plant (g) |
| --- | --- | --- | --- | --- | --- | --- |
| Minimum | 66.00 | 44.00 | 129.00 | 1.00 | 35.00 | 71.00 |
| Maximum | 101.00 | 93.00 | 162.00 | 6.20 | 351.00 | 360.00 |
| Mean | 82.28 | 68.87 | 151.15 | 2.66 | 108.10 | 158.90 |
| SD | 7.87 | 9.92 | 6.01 | 0.71 | 57.56 | 68.40 |
| 10^th^ percentile | 73.00 | 57.80 | 144.80 | 32.00 | 51.60 | 86.00 |
| 2^nd^ quartile | 76.00 | 62.75 | 147.00 | 2.20 | 61.00 | 106.00 |
| Median | 81.00 | 68.00 | 152.00 | 2.60 | 94.50 | 148.50 |
| 3^rd^ quartile | 87.00 | 75.25 | 155.00 | 3.00 | 142.00 | 185.25 |
| 90^th^ percentile | 92.10 | 82.20 | 159.20 | 3.40 | 182.00 | 256.50 |
| CV (%) | 9.56 | 14.40 | 3.98 | 26.63 | 53.25 | 43.05 |

**Table S4**. Statistical data on the variations of anti-nutrient factors across 68 faba bean accessions and between green and yellow genotypes.

| Category | Statistics | Vicine  (mg/g) | Convicine  (mg/g) | TVC  (mg/g) | V:C  ratio | TPC  (mg GAE/g) | TTC  (mg CE/g) | TSC  (mg DE/g) |
| --- | --- | --- | --- | --- | --- | --- | --- | --- |
| Total population | Minimum | 3.41 | 1.75 | 9.19 | 0.42 | 1.65 | 1.70 | 4.12 |
|  | Maximum | 12.64 | 9.74 | 19.34 | 5.67 | 4.38 | 9.47 | 9.50 |
|  | Mean | 9.00 | 5.17 | 14.17 | 2.11 | 2.80 | 4.00 | 6.69 |
|  | SD | 1.62 | 2.23 | 2.78 | 1.02 | 0.56 | 1.48 | 1.22 |
|  | 90^th^ percentile | 10.91 | 8.86 | 18.30 | 3.37 | 3.59 | 5.92 | 8.08 |
|  | 3^rd^ quartile | 10.17 | 6.59 | 17.07 | 2.81 | 3.23 | 5.08 | 7.33 |
|  | Median | 8.92 | 4.73 | 13.37 | 1.91 | 2.73 | 3.63 | 6.91 |
|  | 1^st^ quartile | 7.80 | 3.32 | 12.12 | 1.34 | 2.47 | 2.96 | 5.74 |
|  | 10^th^ percentile | 6.96 | 2.63 | 11.32 | 0.99 | 1.95 | 2.40 | 5.09 |
|  | CV | 18.05 | 16.29 | 19.61 | 48.58 | 20.15 | 37.11 | 18.27 |
| Genotype | Green | 8.80 ± 1.47a | 5.40 ± 2.00a | 14.20 ± 2.78a | 1.85 ± 0.69a | 2.63 0.57a | 4.63 ± 1.67a | 6.52 ±1.31a |
|  | Yellow | 9.13 ± 1.70a | 5.03 ± 2.35a | 14.16 ± 2.78a | 2.27 ±1.15a | 2.91 ± 0.53a | 3.61 ± 1.20b | 6.79 ± 1.22a |

**Table S5**. Variations of nutritional components and antioxidant activities across 68 faba bean accessions genotypes

| Accession | Nutritional components | | | |  | Antioxidant activities | | |
| --- | --- | --- | --- | --- | --- | --- | --- | --- |
|  | DFC  (%) | CFC  (%) | TF  (%) | TP  (%) |  | DPPH  (mg AAE/g) | ABTS  (mg TE/g) | FRAP  (mg AAE/g) |
| VF001 | 16.89 ± 0.89e-m | 8.59 ± 0.03g-h | 1.07 ± 0.10w-ab | 24.86 ± 0.01ah |  | 0.91 ± 0.01n-v | 4.05 ± 0.17p-v | 2.25 ± 0.15d-h |
| VF002 | 16.40 ± 1.89g-o | 8.12 ± 0.19i-k | 0.93 ± 0.04aa-ab | 26.90 ± 0.07w-y |  | 0.63 ± 0.10x-ad | 2.92 ± 0.54y-ab | 2.39 ± 0.15d-e |
| VF003 | 15.74 ± 2.11g-s | 8.41 ± 0.11g-i | 1.30 ± 0.13k-r | 26.17 ± 0.09ad-ae |  | 0.57 ± 0.05aa-ad | 3.26 ± 0.17v-z | 1.84 ± 0.20i-r |
| VF004 | 15.33 ± 0.43g-t | 8.14 ± 0.25i-k | 1.45 ± 0.03h-m | 24.28 ± 0.17ai |  | 2.20 ± 0.25a | 5.26 ± 0.15e-j | 4.94 ± 0.37a |
| VF005 | 14.56 ± 0.72h-t | 6.51 ± 0.05n-o | 0.96 ± 0.02z-ab | 28.45 ± 0.41m-o |  | 0.73 ± 0.07r-ac | 4.77 ± 0.60f-p | 1.90 ± 0.11h-p |
| VF006 | 17.00 ± 2.64e-m | 8.11 ± 0.07i-k | 1.05 ± 0.06w-ab | 26.31 ± 0.11ab-ae |  | 0.82 ± 0.03p-aa | 4.09 ± 0.36n-v | 1.49 ± 0.01q-z |
| VF007 | 17.58 ± 2.57c-i | 8.70 ± 0.11f-g | 1.45 ± 0.09h-m | 28.49 ± 0.27m-o |  | 1.04 ± 0.04k-p | 4.39 ± 0.06k-t | 1.89 ± 0.03h-q |
| VF008 | 13.78 ± 1.44l-u | 7.80 ± 0.17k-l | 1.66 ± 0.15d-f | 21.68 ± 0.09am |  | 1.31 ± 0.13f-j | 5.43 ± 0.47d-h | 2.40 ± 0.20d-e |
| VF009 | 20.06 ± 2.15b-e | 10.85 ± 0.28a | 1.65 ± 0.12d-g | 22.95 ± 0.18ak |  | 1.88 ± 0.13b | 6.91 ± 0.07a-b | 3.25 ± 0.19c |
| VF010 | 16.15 ± 0.81g-p | 8.29 ± 0.00h-j | 1.30 ± 0.07l-r | 28.46 ± 0.12m-o |  | 0.94 ± 0.02m-t | 4.68 ± 0.37g-q | 1.76 ± 0.03k-s |
| VF011 | 15.63 ± 0.87g-s | 9.64 ± 0.11b-c | 1.29 ± 0.02l-s | 22.08 ± 0.14al |  | 0.98 ± 0.06m-s | 5.54 ± 0.07d-f | 1.70 ± 0.18l-u |
| VF012 | 13.34 ± 0.18n-u | 7.74 ± 0.19l | 1.98 ± 0.04a | 21.76 ± 0.19am |  | 2.33 ± 0.01a | 7.26 ± 0.07a | 4.47 ± 0.19b |
| VF013 | 14.86 ± 0.55h-t | 8.54 ± 0.16g-h | 1.41 ± 0.13i-n | 23.30 ± 0.22aj |  | 1.08 ± 0.03j-p | 4.10 ± 0.11n-v | 1.92 ± 0.04g-o |
| VF014 | 16.96 ± 2.37e-m | 9.34 ± 0.19c-d | 1.44 ± 0.11h-m | 27.31 ± 0.11t-v |  | 1.19 ± 0.04h-m | 3.79 ± 0.23s-x | 2.29 ± 0.16d-g |
| VF015 | 12.13 ± 0.28t-v | 9.00 ± 0.28d-f | 1.50 ± 0.05g-j | 21.91 ± 0.16al-am |  | 1.03 ± 0.09k-q | 4.25 ± 0.07m-u | 1.83 ± 0.15j-r |
| VF016 | 12.06 ± 2.58t-v | 7.07 ± 0.15m | 1.10 ± 0.01u-z | 27.52 ± 0.10s-u |  | 1.25 ± 0.01g-l | 4.06 ± 0.08o-v | 1.93 ± 0.05g-n |
| VF017 | 14.30 ± 1.46i-u | 9.31 ± 0.36c-d | 0.95 ± 0.03z-ab | 26.80 ± 0.06x-z |  | 0.92 ± 0.08n-t | 3.84 ± 0.12q-w | 1.51 ± 0.11p-y |
| VF018 | 21.62 ± 0.28b | 9.28 ± 0.21d | 1.35 ± 0.04i-q | 27.56 ± 0.21s-u |  | 1.51 ± 0.06d-f | 4.03 ± 0.03p-v | 2.08 ± 0.04e-l |
| VF019 | 20.27 ± 0.37b-d | 8.15 ± 0.04i-k | 1.46 ± 0.13h-l | 27.00 ± 0.20v-x |  | 1.03 ± 0.08k-q | 3.07 ± 0.09w-aa | 1.59 ± 0.04m-w |
| VF020 | 20.61 ± 0.64b-c | 8.92 ± 0.05e-f | 1.44 ± 0.04h-m | 29.25 ± 0.04i-j |  | 0.97 ± 0.04m-s | 2.73 ± 0.15z-ab | 1.48 ± 0.09r-z |
| VF021 | 12.69 ± 0.15r-u | 8.04 ± 0.10j-l | 1.40 ± 0.01i-n | 28.66 ± 0.08l-n |  | 1.00 ± 0.07l-q | 3.08 ± 0.23w-aa | 1.55 ± 0.08n-x |
| VF022 | 12.79 ± 1.96p-u | 7.98 ± 0.26j-l | 1.13 ± 0.04s-y | 26.74 ± 0.07x-aa |  | 1.51 ± 0.06d-f | 3.47 ± 0.20u-z | 2.44 ± 0.13d-e |
| VF023 | 11.09 ± 0.09u-v | 6.37 ± 0.16o | 1.26 ± 0.12n-u | 30.62 ± 0.07f |  | 0.73 ± 0.04s-ac | 2.43 ± 0.07aa-ac | 0.98 ± 0.07ab-ae |
| VF024 | 12.54 ± 0.32s-u | 9.32 ± 0.10c-d | 1.18 ± 0.04p-x | 25.43 ± 0.04ag |  | 1.16 ± 0.22i-n | 5.33 ± 0.11d-i | 1.66 ± 0.08m-v |
| VF025 | 9.18 ± 0.99v | 8.56 ± 0.41g-h | 1.37 ± 0.10i-o | 25.66 ± 0.10ag |  | 1.42 ± 0.18d-h | 4.58 ± 0.52i-s | 1.97 ± 0.24f-m |
| VF026 | 15.76 ± 1.53g-s | 9.75 ± 0.01b | 1.18 ± 0.06q-x | 28.76 ± 0.07k-m |  | 0.61 ± 0.08z-ad | 3.00 ± 0.26x-ab | 0.77 ± 0.12ad-ae |
| VF027 | 19.72 ± 0.10b-f | 9.05 ± 0.09d-e | 1.20 ± 0.08o-w | 26.55 ± 0.14z-ac |  | 0.53 ± 0.02ab-ad | 2.78 ± 0.15z-ab | 0.67 ± 0.02ae |
| VF028 | 16.50 ± 0.79f-n | 9.33 ± 0.00c-d | 1.00 ± 0.04y-ab | 28.32 ± 0.04n-p |  | 0.64 ± 0.10w-ad | 2.25 ± 0.14ab-ac | 0.75 ± 0.10ad-ae |
| VF029 | 15.30 ± 1.52g-t | 8.99 ± 0.05d-f | 1.31 ± 0.10k-r | 27.26 ± 0.09u-v |  | 1.02 ± 0.13l-q | 4.31 ± 0.31l-u | 1.35 ± 0.20t-ab |
| VF030 | 12.17 ± 1.39t-v | 6.53 ± 0.15n-o | 1.20 ± 0.02p-x | 26.45 ± 0.45aa-ad |  | 1.86 ± 0.27b-c | 6.47 ± 0.82b-c | 2.95 ± 0.33c |
| VF031 | 15.23 ± 1.34g-t | 5.72 ± 0.17p-r | 1.31 ± 0.01k-r | 26.71 ± 0.23x-aa |  | 0.66 ± 0.10u-ad | 4.96 ± 0.19f-m | 1.09 ± 0.17z-ad |
| VF032 | 25.47 ± 0.07a | 9.05 ± 0.15d-e | 1.33 ± 0.08j-q | 25.47 ± 0.20ag |  | 0.88 ± 0.06o-x | 4.18 ± 0.20m-u | 1.08 ± 0.01aa-ad |
| VF033 | 16.51 ± 1.37f-n | 4.82 ± 0.06v-w | 0.95 ± 0.05z-ab | 29.71 ± 0.14g-h |  | 1.59 ± 0.10d-e | 5.85 ± 0.66c-e | 2.61 ± 0.06d |
| VF034 | 16.81 ± 1.60e-m | 3.87 ± 0.06aa-ac | 0.90 ± 0.06ab | 29.30 ± 0.22i-j |  | 0.77 ± 0.04q-ab | 3.28 ± 0.29v-z | 1.50 ± 0.10p-y |
| VF035 | 13.14 ± 2.35o-u | 3.42 ± 0.00ae | 1.03 ± 0.10x-ab | 26.27 ± 0.06ac-ae |  | 1.28 ± 0.11f-k | 5.17 ± 0.44e-k | 2.11 ± 0.13e-k |
| VF036 | 17.13 ± 1.13d-l | 4.44 ± 0.10x-y | 0.92 ± 0.05aa-ab | 28.09 ± 0.05p-r |  | 1.48 ± 0.26d-g | 5.86 ± 0.40c-e | 2.45 ± 0.34d-e |
| VF037 | 13.14 ± 1.41o-u | 5.36 ± 0.18r-t | 1.34 ± 0.03j-q | 25.57 ± 0.09ag |  | 1.15 ± 0.09i-m | 6.08 ± 0.60c-d | 1.74 ± 0.09k-t |
| VF038 | 15.99 ± 1.35g-r | 5.63 ± 0.18q-r | 1.28 ± 0.08m-t | 27.99 ± 0.18p-r |  | 0.92 ± 0.14n-u | 4.46 ± 0.30j-t | 1.69 ± 0.26l-u |
| VF039 | 12.76 ± 0.18q-u | 3.50 ± 0.08ad-ae | 1.88 ± 0.18a-c | 29.05 ± 0.18jk |  | 0.66 ± 0.00v-ad | 4.23 ± 0.33m-u | 1.03 ± 0.01aa-ae |
| VF040 | 17.11 ± 0.46d-m | 4.46 ± 0.10x-y | 1.32 ± 0.07k-q | 25.51 ± 0.31ag |  | 1.05 ± 0.16k-p | 4.90 ± 0.56f-o | 1.64 ± 0.24m-w |
| VF041 | 14.87 ± 0.31h-t | 4.17 ± 0.23y-aa | 1.11 ± 0.02t-z | 34.40 ± 0.16a |  | 0.95 ± 0.08m-t | 5.11 ± 0.46e-l | 1.35 ± 0.11t-ab |
| VF042 | 17.16 ± 0.13d-k | 5.45 ± 0.10q-s | 1.35 ± 0.08j-p | 27.56 ± 0.13s-u |  | 0.91 ± 0.05n-v | 5.46 ± 0.62d-g | 1.37 ± 0.18s-aa |
| VF043 | 14.49 ± 0.36h-t | 5.54 ± 0.26q-s | 1.70 ± 0.14d-e | 27.63 ± 0.19s-t |  | 0.99 ± 0.12m-r | 5.23 ± 0.15e-j | 1.33 ± 0.12u-ab |
| VF044 | 13.00 ± 0.87p-u | 3.84 ± 0.16aa-ad | 1.34 ± 0.04i-q | 28.56 ± 0.26l-n |  | 0.93 ± 0.11m-t | 5.42 ± 0.02d-g | 1.29 ± 0.22u-ab |
| VF045 | 13.38 ± 0.56n-u | 3.67 ± 0.03ab-ae | 1.80 ± 0.17b-d | 26.02 ± 0.05ae-af |  | 1.36 ± 0.05e-i | 5.83 ± 0.15c-e | 2.23 ± 0.04d-i |
| VF046 | 13.84 ± 0.30k-u | 3.90 ± 0.17aa-ac | 1.70 ± 0.12d-e | 26.03 ± 0.26ae-af |  | 0.90 ± 0.14n-w | 4.93 ± 0.52f-m | 1.40 ± 0.18s-aa |
| VF047 | 15.33 ± 1.77g-t | 5.65 ± 0.21p-r | 1.58 ± 0.15e-h | 26.50 ± 0.03z-ac |  | 0.60 ± 0.09z-ad | 2.70 ± 0.23z-ab | 1.13 ± 0.09y-ad |
| VF048 | 13.76 ± 0.27m-u | 5.24 ± 0.16s-u | 1.33 ± 0.02j-q | 27.85 ± 0.17q-s |  | 1.48 ± 0.18d-g | 4.64 ± 0.33g-r | 2.36 ± 0.27d-e |
| VF049 | 13.44 ± 1.95n-u | 5.99 ± 0.25p | 1.51 ± 0.08f-i | 23.31 ± 0.29aj |  | 1.03 ± 0.05k-q | 3.68 ± 0.40t-y | 1.62 ± 0.06m-w |
| VF050 | 17.59 ± 0.74c-i | 6.82 ± 0.04m-n | 1.24 ± 0.08n-v | 28.88 ± 0.30k-l |  | 0.44 ± 0.04ad | 2.81 ± 0.30z-ab | 0.79 ± 0.05ac-ae |
| VF051 | 20.30 ± 2.03b-d | 4.25 ± 0.09y-z | 1.44 ± 0.12h-m | 28.86 ± 0.20k-l |  | 0.62 ± 0.10y-ad | 3.81 ± 0.19r-w | 1.42 ± 0.09s-aa |
| VF052 | 18.32 ± 0.66c-g | 5.45 ± 0.03q-s | 0.92 ± 0.05aa-ab | 31.08 ± 0.12e |  | 0.50 ± 0.01ac-ad | 1.87 ± 0.19ac | 0.86 ± 0.01ac-ae |
| VF053 | 18.48 ± 0.14c-g | 5.72 ± 0.01p-q | 1.43 ± 0.08h-m | 26.62 ± 0.30y-ab |  | 1.39 ± 0.10e-i | 5.90 ± 0.27c-e | 2.20 ± 0.04e-j |
| VF054 | 16.93 ± 0.11e-m | 4.97 ± 0.00u-v | 1.19 ± 0.10p-x | 27.82 ± 0.09r-s |  | 1.37 ± 0.12e-i | 4.19 ± 0.18m-u | 2.19 ± 0.14e-j |
| VF055 | 17.11 ± 0.73d-m | 4.91 ± 0.04u-w | 1.05 ± 0.09w-ab | 25.72 ± 0.06af-ag |  | 0.84 ± 0.04p-z | 4.25 ± 0.26m-u | 1.53 ± 0.10o-x |
| VF056 | 15.54 ± 0.36g-s | 4.25 ± 0.18y-z | 1.06 ± 0.08y-ab | 32.04 ± 0.13d |  | 0.73 ± 0.09s-ac | 4.16 ± 0.30m-u | 1.25 ± 0.20w-ab |
| VF057 | 17.32 ± 0.28d-j | 3.85 ± 0.02aa-ad | 1.00 ± 0.09y-ab | 33.69 ± 0.07b |  | 1.05 ± 0.15k-p | 3.51 ± 0.41u-z | 1.56 ± 0.18n-x |
| VF058 | 14.80 ± 2.41h-t | 4.59 ± 0.01w-x | 1.12 ± 0.07t-z | 26.12 ± 0.22ad-ae |  | 0.97 ± 0.03m-s | 4.62 ± 0.15h-s | 1.59 ± 0.06m-w |
| VF059 | 15.30 ± 1.33g-t | 4.79 ± 0.01v-w | 1.14 ± 0.02r-y | 28.09 ± 0.23p-r |  | 0.87 ± 0.01p-y | 4.07 ± 0.27o-v | 1.27 ± 0.05v-ab |
| VF060 | 15.20 ± 2.72g-t | 3.67 ± 0.14ab-ae | 1.08 ± 0.11v-aa | 30.34 ± 0.18f |  | 0.70 ± 0.01t-ac | 4.56 ± 0.26i-s | 1.17 ± 0.02x-ac |
| VF061 | 15.90 ± 0.78g-s | 3.56 ± 0.16ac-ae | 1.89 ± 0.07a-c | 29.63 ± 0.40g-h |  | 1.65 ± 0.06c-d | 3.92 ± 0.64q-v | 2.33 ± 0.39d-f |
| VF062 | 16.11 ± 2.38g-q | 5.40 ± 0.14q-t | 1.91 ± 0.24a-b | 30.94 ± 0.21e |  | 0.94 ± 0.04m-t | 4.15 ± 0.26m-u | 1.65 ± 0.01m-w |
| VF063 | 14.18 ± 1.35j-u | 3.81 ± 0.10ab-ad | 1.79 ± 0.07b-d | 29.44 ± 0.15h-i |  | 1.14 ± 0.12i-o | 3.84 ± 0.55q-w | 2.06 ± 0.18e-l |
| VF064 | 14.72 ± 1.55h-t | 3.84 ± 0.14aa-ad | 1.74 ± 0.04c-e | 28.18 ± 0.19o-q |  | 1.45 ± 0.23d-g | 4.83 ± 0.45f-p | 2.45 ± 0.41d-e |
| VF065 | 15.76 ± 1.37g-s | 3.98 ± 0.10z-ab | 1.99 ± 0.09a | 27.19 ± 0.17v-w |  | 0.98 ± 0.15m-s | 5.14 ± 0.53e-k | 1.76 ± 0.27k-s |
| VF066 | 15.25 ± 2.21g-t | 5.07 ± 0.22t-v | 1.48 ± 0.05h-k | 29.79 ± 0.15g |  | 0.58 ± 0.04z-ad | 3.78 ± 0.15s-x | 1.04 ± 0.04aa-ae |
| VF067 | 17.76 ± 0.94c-h | 3.72 ± 0.15ab-ae | 1.11 ± 0.01t-z | 31.88 ± 0.25d |  | 0.91 ± 0.06n-v | 3.85 ± 0.42q-w | 1.66 ± 0.03m-v |
| VF068 | 22.63 ± 0.29b | 7.93 ± 0.09k-l | 1.06 ± 0.09w-ab | 32.47 ± 0.26c |  | 0.92 ± 0.06n-u | 5.18 ± 0.30e-k | 1.39 ± 0.10s-aa |

Different unpaired (a-z) or paired (aa-az) letters indicate significantly different mean values in a column (p < 0.05). ABTS: ABTS^•+^ scavenging activity; CFC: crude fiber content; DFC: dietary fiber content; DPPH: DPPH^•^ scavenging activity; FRAP: Ferric reducing power; TF: total fat, TP: total protein.

**Table S6**. Variations of nutritional components, fatty acids, and antioxidant activities between green and yellow genotypes.

| Category | Parameters | Green | | |  | Yellow | | |
| --- | --- | --- | --- | --- | --- | --- | --- | --- |
|  |  | Range | Mean ± SD | CV |  | Range | Mean ± SD | CV |
| Nutritional  components | Crude fiber (%) | 3.5-9.75 | 7.52 ± 1.86a | 24.77 |  | 3.42-10.85 | 5.79 ± 1.99b | 34.44 |
|  | Dietary fiber (%) | 9.18-25.46 | 16.53 ± 3.29a | 19.89 |  | 11.09-22.63 | 15.35 ± 2.46b | 14.89 |
|  | Total fat (%) | 0.93-1.88 | 1.30 ± 0.20a | 15.60 |  | 0.9-1.99 | 1.35 ± 0.32a | 23.66 |
|  | Total protein (%) | 22.08-30.34 | 27.25 ± 1.77a | 6.49 |  | 21.68-34.4 | 27.60 ± 3.05a | 11.04 |
| Fatty acids | Palmitic acid (%) | 15.4-18.73 | 16.97 ± 0.74a | 4.38 |  | 15.13-28.39 | 16.92 ± 1.94b | 11.44 |
|  | Stearic acid (%) | 2.06-3.26 | 2.57 ± 0.26a | 10.31 |  | 1.96-3.29 | 2.56 ± 0.33a | 12.71 |
|  | Oleic acid (%) | 25.54-35.75 | 31.26 ± 2.45a | 7.85 |  | 23.67-40.29 | 30.03 ± 3.44a | 11.44 |
|  | Linoleic acid (%) | 43.02-51.05 | 46.91 ± 1.94b | 4.14 |  | 38.51-54.93 | 48.23 ± 3.21a | 6.65 |
|  | Linolenic acid (%) | 1.85-2.85 | 2.29 ± 0.29a | 12.69 |  | 1.58-2.9 | 2.26 ± 0.27a | 11.97 |
|  | TSFA (%) | 17.96-21.39 | 19.54 ± 0.81a | 4.17 |  | 17.65-30.88 | 19.48 ± 1.93b | 9.90 |
|  | PUFA (%) | 44.98-53.9 | 49.20 ± 2.06b | 4.19 |  | 40.69-57.14 | 50.49 ± 3.32a | 6.57 |
|  | TUFA (%) | 78.61-82.04 | 80.46 ± 0.81b | 1.01 |  | 69.12-82.35 | 80.52 ± 1.93a | 2.40 |
|  | TUFA:TSFA | 3.68-4.57 | 4.13 ± 0.21b | 5.13 |  | 2.24-4.66 | 4.17 ± 0.36a | 8.69 |
|  | DBI | 127.65-136.19 | 131.95 ± 2.05b | 1.55 |  | 114.01-140.84 | 133.27 ± 4.32a | 3.24 |
| Antioxidant  activities | DPPH (mg AAE/g) | 0.44-1.51 | 0.86 ± 0.27b | 31.76 |  | 0.5-2.33 | 1.17 ± 0.40a | 33.96 |
|  | FRAP (mg AAE/g) | 0.67-2.39 | 1.48 ± 0.47b | 32.00 |  | 0.86-4.94 | 1.97 ± 0.80a | 40.80 |
|  | ABTS (mg TE/g) | 2.25-5.54 | 3.83 ± 0.88b | 22.93 |  | 1.87-7.26 | 4.69 ± 1.09a | 23.20 |

ABTS: ABTS^•+^ scavenging activity; CV: Coefficient of variation; DPPH: DPPH^•^ scavenging activity; FRAP: Ferric reducing power; PUFA: Polyunsaturated fatty acid; SD: Standard deviation; TSFA: Total saturated fatty acid; TUFA: Total unsaturated fatty acid.

**Table S7.** Variations of analyzed parameters among faba bean clusters as obtained from HCA.

| Parameter | I | II | III | IV | V | VI | VII |
| --- | --- | --- | --- | --- | --- | --- | --- |
| Days to flowering | 83.12abc | 87.75a | 78.38bc | 75.00c | 78.00bc | 85.86ab | 80.00abc |
| Days from flowering to maturity | 69.35ab | 59.42c | 74.63a | 75.67a | 78.00a | 62.29bc | 74.00a |
| Days to maturity | 152.47ab | 147.17b | 153.00ab | 150.67ab | 156.00a | 148.14b | 154.00ab |
| Number of seeds per pod | 3.12a | 2.48abc | 2.21bc | 2.67abc | 1.90c | 2.83ab | 3.03ab |
| Number of pods per plant | 135.41a | 54.92c | 104.94ab | 86.17bc | 89.50abc | 117.71ab | 108.17ab |
| Seed weight per plant | 175.24a | 142.83a | 159.88a | 128.83a | 110.50a | 182.86a | 115.33a |
| Convicine | 2.94d | 4.51c | 6.19b | 8.55a | 5.32bc | 7.66a | 4.39c |
| Vicine | 9.29bc | 7.59c | 8.81bc | 9.35bc | 11.64a | 10.43ab | 8.84bc |
| Total vicine-convicine | 12.23c | 12.10c | 15.00b | 17.90a | 16.96ab | 18.09a | 13.22c |
| Vicine-to-convicine ratio | 3.35a | 1.76bcd | 1.63bcd | 1.11d | 2.35b | 1.42cd | 2.13bc |
| Total phenol content | 2.73a | 2.65a | 2.96a | 3.15a | 3.42a | 2.62a | 2.57a |
| Total tannin content | 3.73bc | 5.03ab | 2.82c | 3.69bc | 4.25abc | 5.38a | 4.03abc |
| Total saponin content | 6.37a | 6.70a | 6.76a | 6.02a | 6.65a | 7.48a | 6.90a |
| Dietary fiber content | 14.34b | 16.55ab | 16.91ab | 14.64b | 17.70a | 15.45ab | 15.84ab |
| Crude fiber content | 5.20b | 8.41a | 5.64b | 5.59b | 9.50a | 7.82a | 5.87b |
| Total fat content | 1.51a | 1.31ab | 1.15b | 1.27ab | 1.55a | 1.14b | 1.44ab |
| Total protein content | 27.55ab | 26.83ab | 28.54a | 26.81ab | 23.62b | 28.08ab | 27.97ab |
| Palmitic acid | 16.57b | 17.20a | 16.44bc | 15.91c | 16.34bc | 17.55a | 17.17a |
| Stearic acid | 2.46b | 2.48b | 2.52b | 2.54b | 3.02a | 2.45b | 3.03a |
| Oleic acid | 30.41bc | 32.35ab | 29.36c | 33.80a | 32.99ab | 28.47c | 27.88c |
| Linoleic acid | 48.24a | 45.78b | 49.39a | 45.77b | 45.57b | 48.98a | 49.63a |
| Linolenic acid | 2.32ab | 2.18bc | 2.29abc | 1.98c | 2.08bc | 2.55a | 2.29abc |
| Total saturated fatty acid | 19.03b | 19.69a | 18.96b | 18.46b | 19.36ab | 20.00a | 20.20a |
| Polyunsaturated fatty acid | 50.73ab | 47.45c | 51.34a | 48.98bc | 48.36bc | 50.96ab | 51.93a |
| Total unsaturated fatty acid | 80.97a | 80.31b | 81.04a | 81.55a | 80.64ab | 80.00b | 79.80b |
| Double bond index | 133.85a | 130.45b | 135.00a | 131.28b | 130.38b | 134.08a | 134.02a |
| TUFA-to-TSFA ratio | 4.26ab | 4.08bcd | 4.28ab | 4.42a | 4.17bc | 4.01cd | 3.96d |
| DPPH^•^ scavenging activity | 1.16b | 0.96b | 0.96b | 0.91b | 2.04a | 1.01b | 0.90b |
| ABTS^•+^ scavenging activity | 4.59b | 3.61b | 4.50b | 4.25b | 6.09a | 4.13b | 4.45b |
| Ferric reducing antioxidant power | 1.88b | 1.75b | 1.61b | 1.61b | 4.10a | 1.56b | 1.41b |

Different letters indicate significantly different mean values in a row (p < 0.05). TSFA: Total saturated fatty acid; TUFA: Total unsaturated fatty acid.

**Table S8.** Quality of top-performing faba bean accessions.

| Performance parameter | Accessions | IT number | Trait | Value |
| --- | --- | --- | --- | --- |
| Low levels of anti-nutrient factors | VF050 | K195821 | Vicine | 1.96 ± 0.29 mg/g |
|  |  |  | Convicine | 7.23 ± 0.22 mg/g |
|  |  |  | TVC | 9.19 ± 0.28 mg/g |
|  |  |  | Total tannin | 1.96 ± 0.10 mg CE/g |
|  |  |  | Total saponin | 1.70 ± 0.17 mg DE/g |
| High fiber content | VF032 | K152839 | Dietary fiber | 25.47 ± 0.07% |
|  |  |  | Crude fiber | 9.05 ± 0.15% |
|  | VF009 | IT-208235 | Dietary fiber | 20.06 ± 2.15% |
|  |  |  | Crude fiber | 10.85 ± 0.28% |
|  | VF018 | K125306 | Dietary fiber | 21.62 ± 0.28% |
|  |  |  | Crude fiber | 9.28 ± 0.21% |
| High protein content | VF041 | K193648 | Total protein | 34.40 ± 0.16% |
|  | VF057 | K204878 | Total protein | 33.69 ± 0.07% |
| High antioxidant activities | VF012 | IT-208724 | DPPH | 2.33 ± 0.01mg AAE/g |
|  |  |  | ABTS | 7.26 ± 0.07 mg TE/g |
|  |  |  | FRAP | 4.47 ± 0.19 mg AAE/g |
|  | VF004 | IT-188090 | DPPH | 2.20 ± 0.25 mg AAE/g |
|  |  |  | FRAP | 4.94 ± 0.37 mg AAE/g |
| High lipid quality | VF062 | K260906 | Linoleic acid | 52.26 ± 3.38% |
|  |  |  | Linolenic acid | 2.90 ± 0.64% |
|  | VF027 | K125325 | TUFA:TSFA ratio | 3.68 |
|  | VF012 | IT-208724 | DBI | 123.84 |
| Agronomic performance | VF019 | K125310 | DM | 143.00 days |
|  |  |  | SPP (n) | 3.40 |
|  |  |  | SWP | 186.00 g |
|  | VF027 | K125325 | DM | 145.00 days |
|  |  |  | SPP (n) | 2.20 |
|  |  |  | SWP | 360 g |

ABTS: ABTS^•+^ scavenging activity; DBI: Double bond index; DM: Days to maturity; DPPH: DPPH^•^ scavenging activity; FRAP: Ferric reducing antioxidant power; SPP: Number of seeds per pod; SWP: Seed weight per plant; TSFA: Total saturated fatty acid; TUFA: Total unsaturated fatty acid; TVC: Total vicine-convicine content.
